# Supplementary material for: Dissection of two routes to naïve pluripotency using different kinase inhibitors
Source: Nat Commun. 2021 Mar 25;12:1863. doi: 10.1038/s41467-021-22181-5 (PMC7994667; doi:10.1038/s41467-021-22181-5)
Supplement: Supplementary file 1 — Supplementary Information [file 41467_2021_22181_MOESM1_ESM.pdf]

Supplementary material for:

DISSECTION OF TWO ROUTES TO NAÏVE PLURIPOTENCY USING DIFFERENT KINASE INHIBITORS

Ana Martinez-Val[1,2], Cian Lynch[3], Isabel Calvo[3], Pilar Ximénez-Embún[1,4], Fernando Garcia[1,4], Eduardo Zarzuela[1,4], Manuel Serrano[3,5], Javier Munoz[1,4*]

[1] *Proteomics Unit, Spanish National Cancer Research Centre (CNIO), 28029 Madrid, Spain.*

[2] *Current address: The Novo Nordisk Foundation Center for Protein Research, University of Copenhagen, Copenhagen, Denmark*

[3] *Institute for Research in Biomedicine (IRB Barcelona), Barcelona Institute of Science and Technology (BIST)*

[4] *ISCIII-ProteoRed, Spain*

[5] *Catalan Institution for Research and Advanced Studies (ICREA), Barcelona, Spain*

* Correspondence to: jmunozpe@cnio.es

- Supplementary Methods
- Supplementary Figures 1-9
- Supplementary Notes 1-3 and Supplementary Figures 10-12
- Supplementary References

Supplementary Methods

Full proteome Time Course

Sample preparation

The experimental design consisted of four different mouse embryonic stem cell lines: TNGA, TON, ZS and BL6. Each one of the cell lines were grown in vitro in the presence of serum and leukemia inhibitory factor (LIF). Besides, each one of the established cultures were treated with either 2i (GSK3 inhibitor and MEK inhibitor) or Cdk8/19i. As a result, there were four cell lines and three treatments: Serum+LIF (from now on referred as "control"), 2i and Cdk8/19i. Cell pellets were collected by trypsinization at different time points: 1, 2, 4, 7, 10 and 14 days of treatment and washed with PBS and preserved at -80ºC for further analysis.

Cells pellets were lysed using 7 M urea, 2 M thiourea, 50 mM Hepes, 1:1000 (v/v) of benzonase and 1:100 (v/v) of Halt™ phosphatase and protease inhibitor cocktail 100x. Cell lysates were homogenized by vortex and sonication and cleared by centrifugation (20,000 g, 10 min, 4ºC). Protein concentration was measured by Qubit® Protein Assay Kit.

100 µg of each lysate were digested using the filter aided sample preparation (FASP) method [1]. Samples were dissolved in 8M urea and 0.1M TEAB (UTEAB). Proteins were reduced in 15 mM TCEP and alkylated in 30 mM of iodoacetamide for 1 hour in dark, agitated at 300 rpm. Sample was cleaned twice with UTEAB. First digestion using endoproteinase Lys-C (1:50 w/w, Wako Pure Chemical Industries) was performed during 4 hours at room temperature in a wet chamber, followed by a dilution 8-fold in 50 mM TEAB to reduce urea concentration. Second digestion using trypsin (1:100 w/w, Promega) was carried out overnight at 37ºC.

Isobaric labelling

The complete labelling scheme is shown in Supplementary Figure 1. For labelling, 100 µg of digested peptides were used for each channel using the iTRAQ® Reagent 8plex kit (AB Sciex). The clean-up was performed with C18 Sep-Pack. Sample was loaded in 1% TFA, washed with 0.2% TFA and finally eluted with 1ml of 70% of acetonitrile and 0.1% of TFA. Eluate was dried in vacuum and dissolved in 10 mM of NH_4OH for subsequent fractionation by high pH reversed phase chromatography.

High pH reverse phase fractionation

Each iTRAQ labelled sample was fractionated by high pH reverse phase chromatography. Peptides were dissolved in 100 µl pf phase A (10 mM NH_4OH). Peptides were eluted at a flow rate of 100 µl/min onto a XBridge® Peptide BEH C18 (3.5 µm, 2.1 x 150 mm) column (Waters) during 60 minutes, using the following gradient of phase B (10 mM NH_4OH, 90% CH_3CN): 0-50 min 25% B, 50-54 min 60% B, 54-61 min 70% B. Samples were collected each minute from minute 10 to minute 55, and concatenated to 20 fractions. Fractions were dried in vacuum and dissolved in 50 µl of 1% formic acid for subsequent LC-MS/MS analysis.

LC-MS/MS

The Impact (Bruker Daltonics) was coupled online to a nanoLC Ultra system (Eksigent), equipped with a CaptiveSpray nanoelectrospray ion source supplemented with a CaptiveSpray nanoBooster operated at 0.2 bar/minute with isopropanol as dopant. Three micrograms of each fraction were loaded onto a reversed-phase C18, 5 µm, 0.1 x 20 mm trapping column (NanoSeparations) and washed for 15 min at 2.5 µl/min with 0.1% FA. The peptides were eluted at a flow rate of 300 nl/min onto an analytical column packed with ReproSil-Pur C18-AQ beads, 2.4 µm, 75 µm x 50 cm (Dr. Maisch Gmb) , heated to 45 °C. Solvent A was 4% ACN in

0.1% FA and Solvent B acetonitrile in 0.1% FA. Peptides were separated using the following gradients: (i) 0–2 min 2% B, 2–119 min 2–20% B, 119–129 min 20-34% B, 129-140 min 98% B, 140–145 min 2% B and (ii) 0-5 min 2% B, 5-12.8 min 3.2% B, 12.8-25.7 min 8.4% B, 25.7-40.75 min 10% B, 40.75-56 minm 12% B, 56-71 min 13.6% B, 71-86.2 min 16% B, 86.2-101.5 min 18.4% B, 101.5-116.5 min 21.1% B, 116.5-131.5 min 24.4% B, 131.5-146.5 min 29.3% B, 146-150min 33.3% B, 150-156min 40%B, 156-172min 98%B, 172-180min 2% B.
The MS acquisition time used for each sample was either 145 or 180 minutes depending on the gradient. The Q-q-TOF Impact was operated in a data dependent mode. The spray voltage was set to 1.35 kV (1868 nA) and the temperature of the source was set to 180ºC. The MS survey scan was performed at a spectra rate of 2.5 Hz in the TOF analyzer scanning a window between 80 and 1600 m/z. The minimum MS signal for triggering MS/MS was set to a normalized threshold of 500 counts. The 20 most abundant isotope patterns with charge ≥2 and m/z > 350 from the survey scan were sequentially isolated and fragmented in the collision cell by collision induced dissociation (CID) using a collision energy of 23 – 56 eV as function of the *m/z* value. The *m/z* values triggering MS/MS with a repeat count of 1 were put on an exclusion list for 30 s using the *rethinking* option: the precursor intensities were re-evaluated in the scan (n) regarding their values in the previous scan (n-1). Any *m/z* whit intensity exceeding 5 times the measured value in the preceding survey scan was reconsidered for MS/MS.

Data processing

Raw files were analyzed using MaxQuant 1.5.3.30 [2] with Andromeda [3] as the search engine against a Mus musculus database (UniProtKB/Swiss-Prot, 43,539 sequences). Sample quantification type was set to iTRAQ8plex. Carbamidomethylation of cysteine was included as fixed modification and oxidation of methionine, acetylation of protein N-terminal were included as variable modifications. Precursor mass tolerance was 35 ppm for the first search, and 7 ppm for the main search. Fragment mass tolerance was set to 40 ppm. Minimal peptide length was set to 7 amino acids and a maximum of two missed-cleavages were allowed. Peptides were filtered at 1% FDR. For protein assessment (FDR <1%) in MaxQuant, at least one unique peptide was required for identification. Only unique peptides were used for protein quantification. Other parameters were set as default. Reverse, contaminants and proteins that did not have reporter intensity in all eight channels were discarded for further analysis.

Reporter intensities of the proteins were extracted from proteinGroups.txt table, transformed to log2 and normalized within each experiment using Loess function from limma [4] package in R. In order to integrate the eight independent experiment a further inter-experiment normalization was conducted using ComBat function from sva [5] package in R.

Statistical determination of differential proteins at each time point versus Serum/LIF initial conditions was performed using limma [4] (two-sided) approach implemented in Prostar (version 1.12.4)[6]. We set a minimum fold change of 0.1 (log2) and a maximum p-value for each condition. The p-value threshold was adjusted by Benjamini-Hochberg correction to a 5% FDR.

Phosphoproteome time Course

Sample preparation

Cell pellets were resuspended in 5% SDS, 50mM triethylammonium bicarbonate buffer supplemented with 1:1000 (v/v) of benzonase and 1:100 (v/v) of Halt™ phosphatase and protease inhibitor cocktail 100x. Samples were lysed at 99ºC during 10 minutes in agitation (1250 rpm). Cell lysates were homogenized by vortexing and sonication and cleared by centrifugation (10,000 g, 10 min). Protein amount was quantified using Nanodrop at 280nm.

Proteins were reduced in 15 mM TCEP and alkylated in 30 mM of chloroacetamide for one hour in dark, agitated at 300 rpm.

A pool sample was generated to include in each labeling experiment by mixing serum/LIF sample from each one of the cell lines used in the experiment.

Two hundred micrograms of protein from each sample were digested using the S-Trap (ProtiFi) method[7]. Two aliquots of 200 µg of control samples were digested separately and in parallel to asses for technical variability that could arise due to sample preparation. Digestion was performed using trypsin (1:50 w/w, Promega) during one hour at 47ºC.

Isobaric labelling

The following labelling scheme was used: 126 pool sample, 127N and 127C serum/LIF or control samples, 128N, 128C, 129N and 129C for 2i treatment at 30 minutes, 1, 2 and 6 hours; 130N, 130C, 131N and 131C for Cdk8/19i treatment at 30 minutes, 1, 2 and 6 hours.

For labelling 200 µg of digested peptides optimized amount of TMT 11plex™ reagent set (Thermo Fisher) were used. Peptides were resuspended in 200 ul of 200mM Hepes (pH 8.5) and the labelling reaction was carried out following manufacturer instructions. Finally, reaction was quenched with 4ul of 5% hydroxylamine during 15 minutes at room temperature and samples were mixed.

The clean-up was performed with C18 Sep-Pack. Sample was loaded in 1% TFA, washed with 0.2% TFA and finally eluted with 700 µl of 80% of acetonitrile and 0.1% of TFA.

Phosphopeptide enrichment

To perform phosphopeptide enrichment, 240 µl of CH_3CN and 60 µl of TFA was added to the eluate of the C18 Sep- Pack. As a result sample will be dissolved in 80% CH_3CN and 6% TFA. Titanium dioxide (TiO_2) beads were preconditionated by washing twice with 1ml of CH_3CN and twice with 500 µl of DHB solution (20 mg/ml DHB in 80% CH_3CN 6% TFA). Finally beads were suspended to a final concentration of 60 µg/µl of DHB solution. TiO_2 beads were added to the sample in a ratio 1:2 (Sample:TiO_2). Sample was incubated in rotation for 15 minutes covered with foil. Afterwards, sample was centrifuged 1 minute at 5000 g. Supernatant was used for a second TiO_2 binding. This time, half of the amount of TiO_2 beads were used for incubation.

Next, beads from the first and second TiO_2 binding were transfer to separate C8-tips and washed with 100 ul pf 10% CH_3CN 6% TFA, 40% CH_3CN 6% TFA and 60% CH_3CN 6% TFA. Peptides were eluted with 25 µl of 5% NH_4OH and 25 µl of 10% NH_4OH 25% CH_3CN. Samples were dried in vacuum up to 5 µl.

Eluate from the second TiO_2 binding was desalted with C18 stage tips, dried and resuspended in 22 µl 5% FA for subsequent LC-MS/MS analysis.

Micro high pH reverse phase fractionation

45 µl of phase A (20 mM NH4OH) was added to the sample obtained from the first TiO_2 incubation. 5 discs of C18 stage tip were used. Sample was loaded into the tips thrice and the flow-through was collected to a vial. Next, 50 µl of phase A was loaded and collected in the same vial as the flow-through. Peptides were subsequently eluted increasing the percentage of Buffer B (20mM NH3 in CH3CN) (i.e. 4, 8, 12, 20, 60 and 80%) of Buffer B. Last three fractions were pooled together. Samples were suspended in 22 µl 5% FA for subsequent LC-MS/MS analysis.

LC-MS/MS

Each fraction were separated by on-line reversed-phase nanoscale capillary LC and analyzed by electrospray MS/MS. The experiments were performed on an Ultimate 3000 RSL nano LC system (Thermo Scientific, Bremen) coupled to an QExactive HF-X mass spectrometer (Thermo Scientific, Bremen) equipped with an EASY-spray ion source (Thermo Scientific, bremen). In order to pre-concentrate and desalt the samples before switching the pre-column in line with the separation column, 9 µL from each sample was loaded onto a nano trap column (100 µm i.d x 2 cm) packed with Acclaim PepMap100 C18 5 µm, and washed for 4 min at 5 µL/min with loading buffer (0.1% FA). The peptides were eluted from an Easy-Spray Column (75 µm i.d. x 50 cm) packed with PepMap RSLC C18 2 µm by application of a binary gradient consisting of 0.1% FA (buffer A) and 100% ACN in 0.1%FA (buffer B), with a flow rate of 250 nL/min during 90 minutes. The column was operated at a constant temperature of 45ºC.

The QExactive HF-X was operated in positive ionization mode. The MS survey scan was performed in the Orbitrap scanning a window between 350 and 1500 *m/z*. The resolution was set to 60,000 FWHM at m/z 200. The *m/z* values triggering MS/MS with a repeat count of 1 were put on an exclusion list for 45 s. The minimum MS signal for triggering MS/MS was set to 1e5. The 15 most abundant isotope patterns with charge ≥2 and <6 from the survey scan were selected with an isolation window of 1 m/z and fragmented in the HCD collision cell. Normalized collision energy was set to 35. The resulting fragments were detected in the Orbitrap scanning a window between 100 and 2000 m/z with a resolution of 45,000 FWHM at *m/z* 200. The maximum ion injection times for the survey scan and the MS/MS scans were 45 ms and 80 ms respectively and the ion target values were set to 5e4 and 8e3, respectively for each scan mode.

Data analysis

Raw files were analyzed using MaxQuant 1.6.0.16 [2] with Andromeda [3] as the search engine against a Mus musculus database (UniProtKB/Swiss-Prot, 43,539 sequences). Sample quantification type was set to TMT11Plex. Carbamidomethylation of cysteine was included as fixed modification and oxidation of methionine, acetylation of protein N-terminal and phosphorylation of serine, threonine and tyrosine were included as variable modifications. Precursor mass tolerance was 20 ppm for the first search, and 4.5 ppm for the main search. Fragment mass tolerance was set to 20 ppm. 17 top peaks were selected per 100 Da interval of the fragmentation spectra. Minimal peptide length was set to 7 amino acids, maximum peptide mass was set to 6500 Da and maximum of two missed-cleavages were allowed. Peptides were filtered at 1% FDR. Other parameters were set as default. Reverse, contaminants and identifications that did not have reporter intensity in all eleven channels were discarded for further analysis.

Reporter intensities of the phosphorylation sites were extracted from Phospho(STY)Sites.txt table. Phospho(STY)Sites.txt table were expanded using Perseus, reporter intensities were transformed to log2 and normalized within each experiment using Loess function from Limma[4] package in R. In order to integrate the eight independent experiment a further inter-experiment normalization was conducted using ComBat function from sva[5] package in R.

Statistical determination of differential phosphorylation sites at each time point versus Serum/LIF initial conditions was performed using limma[4] (two-sided) approach implemented in Prostar (version 1.12.4)[6]. We set a minimum fold change of 0.1 (log2) and a maximum p-value for each condition. The p-value threshold was adjusted by Benjamini-Hochberg correction to a 5% FDR.

Metabolomics

Sample preparation

Metabolomic profiling was performed in collaboration with Metabolon Inc. Samples were prepared using the automated MicroLab STAR® system from Hamilton Company. Several recovery standards were added prior to the first step in the extraction process for QC purposes. To remove protein, dissociate small molecules bound to protein or trapped in the precipitated protein matrix, and to recover chemically diverse metabolites, proteins were precipitated with methanol under vigorous shaking for 2 min (Glen Mills GenoGrinder 2000) followed by centrifugation. The resulting extract was divided into five fractions: two for analysis by two separate reverse phase (RP)/UPLC-MS/MS methods with positive ion mode electrospray ionization (ESI), one for analysis by RP/UPLC-MS/MS with negative ion mode ESI, one for analysis by HILIC/UPLC-MS/MS with negative ion mode ESI, and one sample was reserved for backup. Samples were placed briefly on a TurboVap® (Zymark) to remove the organic solvent. The sample extracts were stored overnight under nitrogen before preparation for analysis.

LC-MS/MS

All methods utilized a Waters ACQUITY ultra-performance liquid chromatography (UPLC) and a Thermo Scientific Q-Exactive high resolution/accurate mass spectrometer interfaced with a heated electrospray ionization (HESI-II) source and Orbitrap mass analyzer operated at 35,000 mass resolution. The sample extract was dried then reconstituted in solvents compatible to each of the four methods. Each reconstitution solvent contained a series of standards at fixed concentrations to ensure injection and chromatographic consistency. One aliquot was analyzed using acidic positive ion conditions, chromatographically optimized for more hydrophilic compounds. In this method, the extract was gradient eluted from a C18 column (Waters UPLC BEH C18-2.1x100 mm, 1.7 µm) using water and methanol, containing 0.05% perfluoropentanoic acid (PFPA) and 0.1% formic acid (FA). Another aliquot was also analyzed using acidic positive ion conditions, however it was chromatographically optimized for more hydrophobic compounds. In this method, the extract was gradient eluted from the same afore mentioned C18 column using methanol, acetonitrile, water, 0.05% PFPA and 0.01% FA and was operated at an overall higher organic content. Another aliquot was analyzed using basic negative ion optimized conditions using a separate dedicated C18 column. The basic extracts were gradient eluted from the column using methanol and water, however with 6.5mM Ammonium Bicarbonate at pH 8. The fourth aliquot was analyzed via negative ionization following elution from a HILIC column (Waters UPLC BEH Amide 2.1x150 mm, 1.7 µm) using a gradient consisting of water and acetonitrile with 10mM Ammonium Formate, pH 10.8. The MS analysis alternated between MS and data-dependent MS^n scans using dynamic exclusion. The scan range varied slighted between methods but covered 70-1000 m/z.

Data extraction and Compound Identification

Raw data was extracted, peak-identified and QC processed using Metabolon’s hardware and software. These systems are built on a web-service platform utilizing Microsoft’s .NET technologies, which run on high-performance application servers and fiber-channel storage arrays in clusters to provide active failover and load-balancing. Compounds were identified by comparison to library entries of purified standards or recurrent unknown entities. Metabolon maintains a library based on authenticated standards that contains the retention time/index (RI), mass to charge ratio (*m/z)*, and chromatographic data (including MS/MS spectral data) on all molecules present in the library. Furthermore, biochemical identifications are based on three

criteria: retention index within a narrow RI window of the proposed identification, accurate mass match to the library +/- 10 ppm, and the MS/MS forward and reverse scores between the experimental data and authentic standards. The MS/MS scores are based on a comparison of the ions present in the experimental spectrum to the ions present in the library spectrum. While there may be similarities between these molecules based on one of these factors, the use of all three data points can be utilized to distinguish and differentiate biochemicals. More than 3300 commercially available purified standard compounds have been acquired and registered into LIMS for analysis on all platforms for determination of their analytical characteristics. Additional mass spectral entries have been created for structurally unnamed biochemicals, which have been identified by virtue of their recurrent nature (both chromatographic and mass spectral). These compounds have the potential to be identified by future acquisition of a matching purified standard or by classical structural analysis.

Metabolite quantification, data normalization and statistical analysis

Peaks were quantified using area-under-the-curve. For studies spanning multiple days, a data normalization step was performed to correct variation resulting from instrument inter-day tuning differences. Essentially, each compound was corrected in run-day blocks by registering the medians to equal one (1.00) and normalizing each data point proportionately (termed the "block correction"). For studies that did not require more than one day of analysis, no normalization is necessary, other than for purposes of data visualization. However, in this experiment we have applied an additional normalization factor based on the total protein as determined by Bradford assay to account for differences in metabolite levels due to differences in the amount of material present in each sample. One of the samples was discarded in this step due to the high presence of missing values (cell line V6.4, treatment 2i, replicate 2).

Afterwards, data processing was performed within Prostar R package[6] (version 1.12.16). Metabolites that were not quantified in at least 75% of the samples in one treatment were discarded. Data was transformed to log2 scale and missing values were imputed using Imp4p for values missing at random, and with LAPALA with upper bound 2.5 for values missing in an entire condition. Once missing values were imputed, data was normalized by quantile centering to the median. Finally, with these data we performed differential analysis using limma (v3.36.1) (two-sided) with a design that account for biological replicates of the same cell line, with p-value correction of 5% FDR.

Z-scoring and Temporal trend classification

Z-score or mean-centering normalization was applied to normalize protein and phosphosite intensities. Z-score was applied after collapsing all replicates to their average at each time point. Next, the proteins differentially expressed at least at one time point in our treatment were clustered using k-means. Prior to this, Gap-statistics was employed to estimate the optimal number of clusters to classify the data. Gap-statistics was implemented in R in the function "clusGap" from the library "cluster", using 400 iterations, a maximum of 30 clusters, k-means algorithm and the "firstmax" method to retrieve the first local maximum. The optimal number of clusters provided by "clusGap" was therefore used to calculate the k-means clusters using the function "cluster_analysis" from the library "multiClust" in R.

RNA- protein comparison

Due to the ratio compression expected in isobaric labelling, in order to compare transcriptomics changes against proteomics changes, log2 fold changes were adjusted using the function "width adjustment" in Perseus.

Functional annotation

ClueGO

After classifying the different kinetics, we extracted biological meaningful information from each group of proteins by performing functional annotation in ClueGO[8]. We proceeded to annotate functionally those groups using Gene Ontology terms (BP, MF and CC) as well as Reactome pathways. ClueGO performs enrichment tests with FDR correction (Benjamini-Hochberg) to select those terms significantly overrepresented within a selected cluster. Also, it fuses terms that share similar associated genes to reduce redundancy. In-house RNASeq data containing all genes transcriptionally active in mESCs was used as a reference set for over-representation analyses.

Motif annotation in Perseus

Potential kinase motifs were annotated using Perseus for class I phosphorylation sites. Fisher's exact test was performed to determine which motifs were significantly represented in each cluster. The background used for the enrichment analysis was comprised of all the class I phosphosites in the data (13,593 sites). A motif was considered significantly over-represented in the FDR was below 0.01 and the enrichment factor was over 1.3. Additionally to the motifs provided by Perseus, we included motifs for ERK, GSK3 and Cdk8 extracted from literature.

Fixation Protocol for Cell Pellet Transmission Electron-Microscopy of mitochondria

Cells were harvested to single cell suspension in a 50ml tube to make a 50ul cell pellet volume at least. Cells were washed with 2x in 1xPBS cold (Resuspended in 1xPBS, and immediately spun down the cells, 300g/2mins, keeping the cells in 1xPBS for the minimal amount of time), and moved to a 1.5ml eppendorf tube. The cells were washed in 1 x in Phosphate Buffer cold (Phosphate Buffer "PB" 0,1M pH 7.4) (Resuspended cells in 1xPB, and immediately spun down, 300g/2mins, keeping the cells in 1xPB for the minimal amount of time). The cells were resuspend in Fix buffer (2%Paraformaldehyde, 2,5% glutaraldehyde in Phosphate Buffer PB 0,1M pH 7.4), using 16% EM-quality para-Formaldehyde stock, and incubated on ice for 10-30mins. The cells were spun down (500g/2mins), supernatant was aspirated, and the cell pellet was resuspended fresh Fix Buffer.

The cell suspension was stored at 4C until processing for electron microscopy with 1% osmium tetraoxide and 0.8% potassium ferrocyanide for a time of between 1h and 2h at 4ºC. The cells were washed with MilliQ water (4 or 5 washes of 10 minutes at 4ºC), and a dehydration sequence was started, with acetone (at 50% for 10min; 70% overnight; and then 10 minutes each for 80%, 90%, 96%, and finally 100% at 4ºC. Cell mounting was at room temperature in Spurr epoxy resin, in the following sequence of Acetone:Spurr epoxy resin: 3 volumes acetone: 1 volume Spurr epoxy resin (overnight); 1 volume acetone: 1 volume Spurr epoxy resin (6h); 1 volume acetone: 3 volumes Spurr epoxy resin (overnight); and then pure Spurr epoxy resin (3 changes: one of 6h, one overnight and one of 4h).

The blocks containing the cells were made and left at least 48-72h in an oven at 80ºC. The semi-fine cuts (survey sections) were continued using a glass blade using a Leica ultramicrotome. The area where the ultra-fine cuts were to be made were selected (using an Olympus

microscope). The ultramicrotomy was performed with a diamond blade (Diatome) and the same ultramicrotome collecting the cuts of about 70nm thick in copper grids of 100, 200 or 400 mesh, and the grid was previously coated with formvar –(formvar-coated copper grids). Ultra-fine cuts were contrasted with uranyl acetate and lead citrate. Observations were made on a TEM JEOL J1010 (tungsten filament), using a Gatan Orius CCD camera with Digital Micrograph software, also from Gatan.

Flow cytometry

MitoTracker staining was employed to analyse mitochondrial mass by flow cytometry. mESCs long-term adapted to the indicated culture conditions were stained with MitoTracker Green FM (Cat no. M7514) and MitoTracker Red CMXRos (Cat no. M7512) for 30 min at 37ºC, at concentrations of 200 nM.

Cells were resuspended in FACs Buffer (1x phosphate-buffered saline (PBS); 0.5% bovine serum albumin (BSA); 1mM Ethylenediaminetetraacetic acid (EDTA)) and DAPI was used to asess cell death. Fluorescence was detected using the filters FL1, FL3 and FL9 as indicated.

ATP assay

ATP activity was assayed using the CellTiter-Glo™ Luminescent Cell Viability Assay (Promega) in accordance with the manufacturer's directions. Luminescence was read on an Orion microplate luminometer (Berthold Detection System GmbH, Oak Ridge, TN, USA). Cells were pretreated with ROTENONE (R8875) at a gradient concentration (0,025 to 0,75uM) for 24h. ATP measurements were run in triplicate; calculations were based upon a standard curve and expressed as percentage of ATP in non-treated wells.

Cell immunofluorescence

mESCs (E14 cell line) were grown on chamber slides using the indicated culture conditions (S/L, or 48 hours of treatment with either 2i or Cdk8/19i). In addition, to aid cell attachment to the slides, slides were pre-treated for 30 minutes with fibronectin 5µg/mL (R&D Systems #1918-FN-02M) diluted in PBS. Cells were fixed 24 hours after plating on chamber slides with 4% paraformaldehyde for 3 minutes at room temperature, washed with PBS and permeabilized with PBS containing for 1 hour. Cells were blocked in 10% FBS/1xPBS for 1h and incubated with antibodies (Mu anti-ERF 1/250, Santa Cruz sc-398269) at 1:500 to 1:1000 in PBS-4%BSA, for 3 hours at 37oC or overnight at 4oC, washed with PBS and further incubated with secondary anti-rabbit or anti-mouse antibodies conjugated with Alexa-488 (Thermo Fisher Scientific, catalog # A-11029 and catalog # A-11034). (1:500 in PBS-4%BSA). Nuclei were counter-stained with DAPI.

Supplementary Figures

Supplementary Figure 1

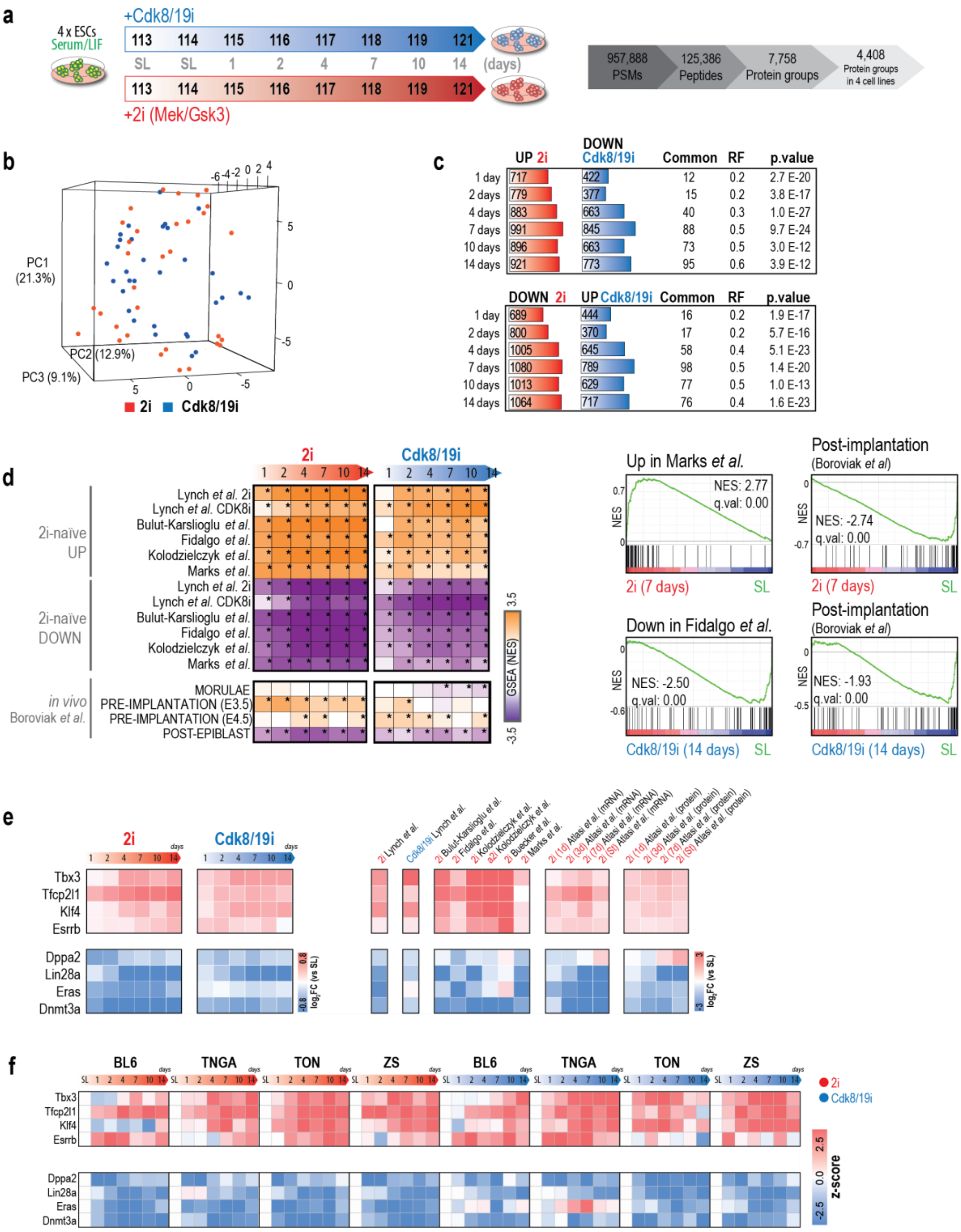

(A) Experimental design, labelling scheme and identification summary from all four iTRAQ8plex experiments.

(B) PCA of the samples treated with 2i (red) and Cdk8/19i (blue).

(C) Differentially regulated proteins (limma, two-sided, FDR <5%) at each time point in 2i and Cdk8/19i. RF indicates the "Representation factor" of the overlap and the p-value the significance of that overlap calculated by an hypergeometric test.

(D) Heatmap with the Normalized Enrichment Score of different gene-sets obtained from the indicated published data-sets: Lynch et al[9], Bulut-Karslioglu et al[10], Fidalgo et al[11], Kolodziejczyk et al[12] and Marks et al[13]. Gene sets contain up and down-regulated genes in mESCs in the naïve state of pluripotency (treated in vitro with 2i and compared to Serum/LIF conditions). Below, GSEA analysis with in vivo mouse embryonic developmental stages obtained from Boroviak et al[14]. Orange indicates over-expression and purple indicates repression. Statistical significance values were calculated using Kolmogorov-Smirnov statistics implemented in the GSEA algorithm. P-values were corrected for multiple testing. An asterisk indicates q.value <0.01. Source data are available as Source Data file.

(E) Fold-changes (log2 scale) for selected markers of naive and primed pluripotency states in our proteomic data sets and in other published mRNA and protein data sets, as indicated.

(F) Changes in protein expression (z-score normalized) in each of the four cell lines analyzed in this study for proteins involved in pluripotency shown in Figure 1D.

Supplementary Figure 2

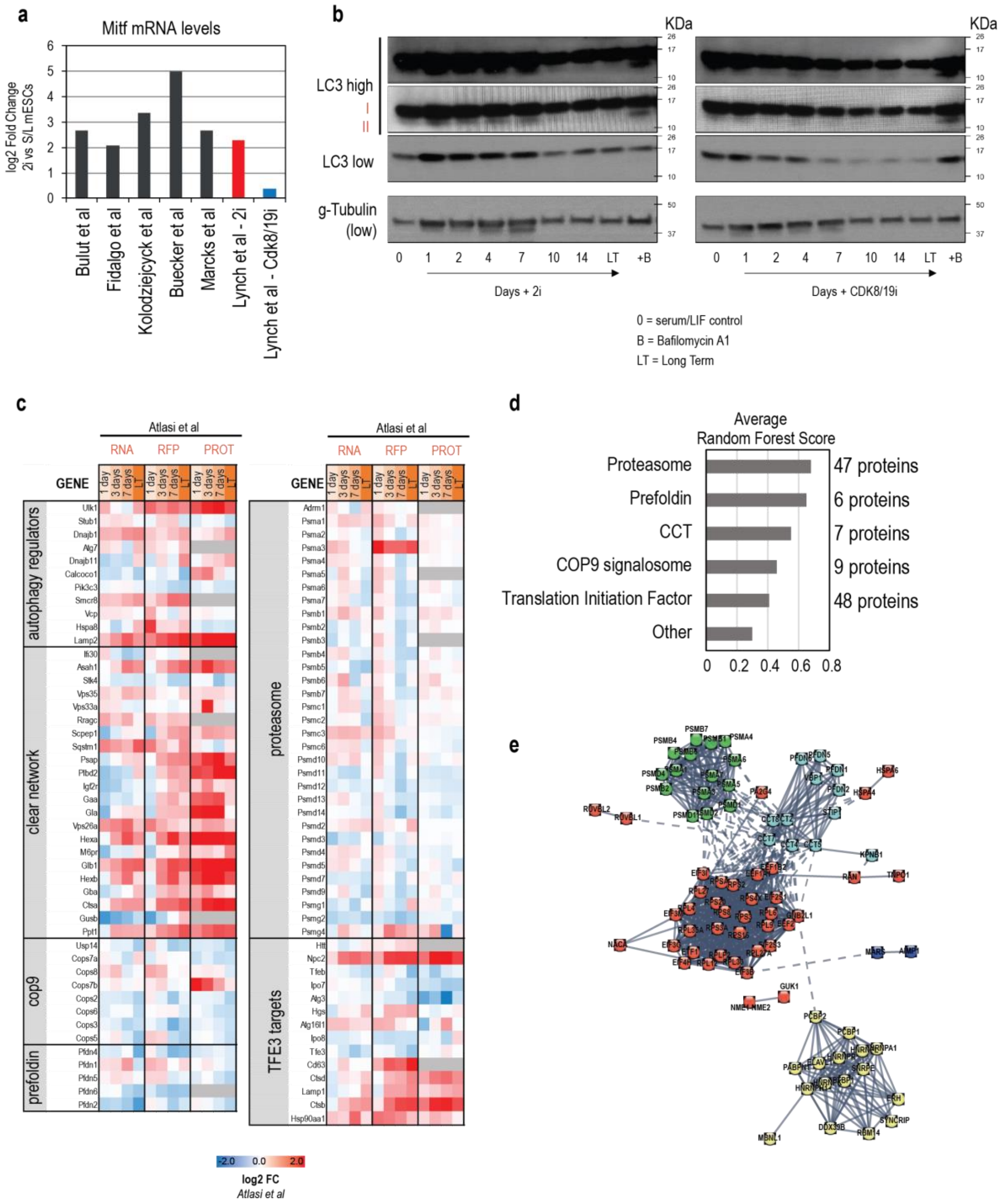

(A) Mitf mRNA log2 fold change of 2i vs Serum/LIF from selected published datasets: Lynch et al[9], Bulut-Karslioglu et al[10], Fidalgo et al[11], Kolodziejczyk et al[12], Buecker et al[17] and Marks et al[13].

(B) Western Blot for LC3B in mESCs treated with 2i (left) o Cdk8/19i (right) in one mES cell line (V6.4) across 8 different days of treatment. The increased levels of LC3-II are observed in several time-points. Source data are available as Source Data file.

(C) Proteins involved in autophagy and proteostasis. Log2 fold change between 2i and S/L conditions for the data from Atlasi et al[15].

(D) Progulon finder results from ProteomeHD using the Proteasome Complex as query. Bars represent the average random forest score obtained for different groups of proteins, as well as the number of hits for each group. Source data are available as Source Data file.

(E) Network representation of proteins co-regulated with the subunit Psma1 of the proteasome calculated using ProteomeHD. Color coding indicates belonging to a cluster, calculated using k-means in STRING. Connection between nodes in based on the confidence of the interaction (minimum required interaction score 0.9).

Supplementary Figure 3

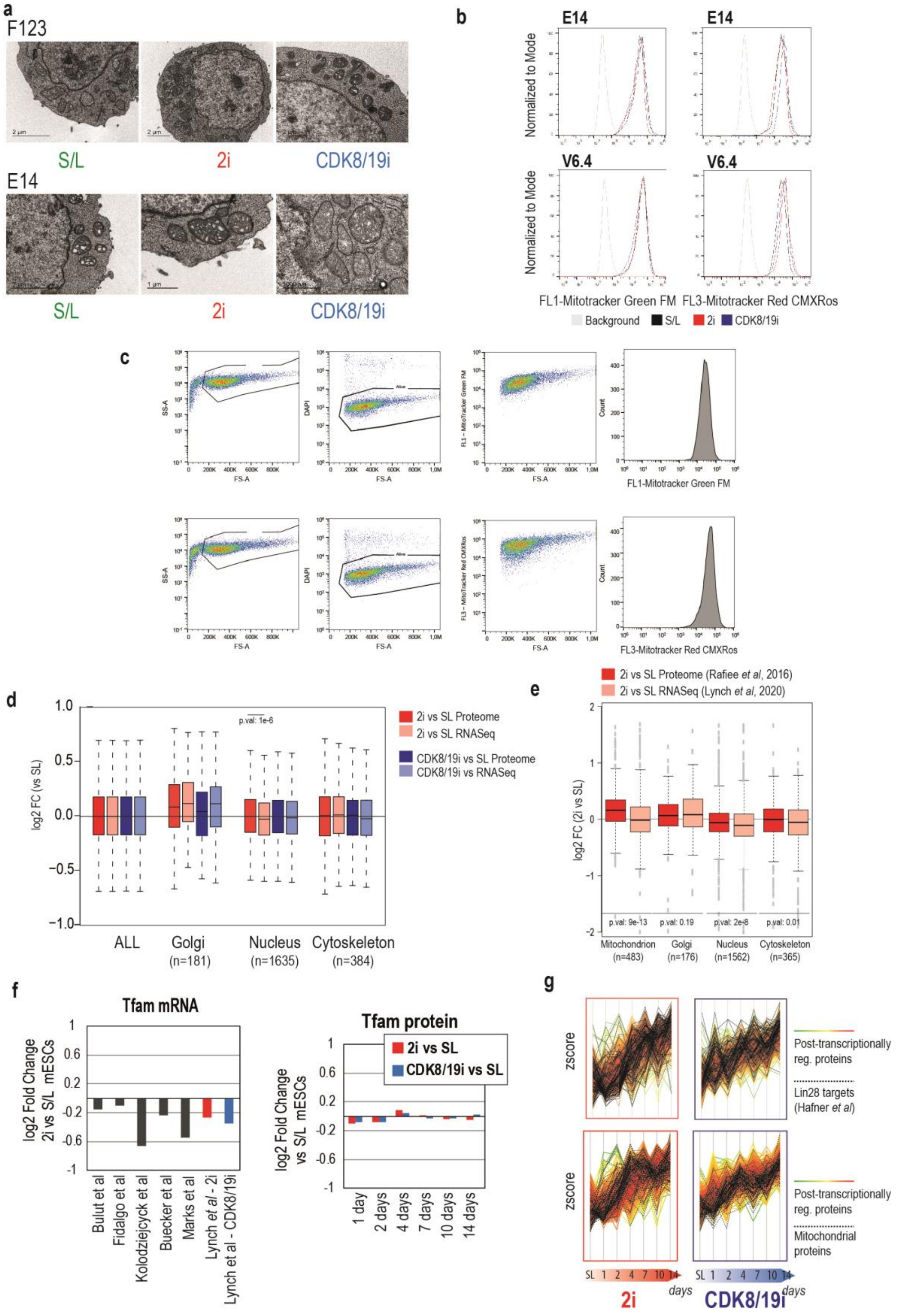

(A) Transmission electron miscrocopy images of mESCs and their mitochondria grown in S/L, 2i and Cdk8/19i in F123 and E14 cell lines showing similar mitochondrial structures in a number of cells (at least 10 cells were imaged per condition).

(B) Mitotracker results for mESCs grown in S/L, 2i and Cdk8/19i.

(C) Schematic showing example of FACS gating strategy in this study for the Mitotracker analysis in Suppl. Fig 3B. DAPI was added to live cell suspension 2mins before analysis, as a live/dead discriminator. Top: example of MitoTracker Green FM staining. Bottom: example of MitoTrackerRed CMXRos staining.

(D) Boxplot of ratios of proteins and mRNAs (total proteome and proteins from the Golgi apparatus, the nucleus and the cytoskeleton) in 2i or Cdk8/19i vs Serum/LIF grown mESCs. For boxplot representations, center lines show the medians; box limits indicate the 25th and 75th percentiles as determined by R software; whiskers extend 1.5 times the interquartile range from the 25th and 75th percentiles, outliers are represented by dots. Statistical analysis was done using a paired two-sample t-test for means, two-sided (N=number of proteins used for the test in each cellular location is shown). Protein ratios are calculated from N=4 independent cell lines. mRNA ratios are calculated from N=3 biological replicates. Source data are available as Source Data file.

(E) Boxplot with the distribution of ratios of proteins from different subcellular compartments in 2i+LIF vs Serum grown mESCs from Rafiee et al[16]. P-value indicates the significance of the difference between both distributions calculated from a two samples t-test. For boxplot representations, center lines show the medians; box limits indicate the 25th and 75th percentiles as determined by R software; whiskers extend 1.5 times the interquartile range from the 25th and 75th percentiles, outliers are represented by dots. Source data are available as Source Data file.

(F) Fold change (2i vs S/L or Cdk8/19i vs S/L) of TFAM mRNA in our data and several published data sets: Lynch et al[9], Bulut-Karslioglu et al[10], Fidalgo et al[11], Kolodziejczyk et al[12], Buecker et al[17] and Marks et al[13]. (left plot). TFAM protein fold changes (2i vs S/L and Cdk8/19i vs S/L) from 1 to 14 days of treatment (right plot).

(G) Profile plots of post-transcriptionally regulated proteins and highlighted in black Lin28 targets[18] and mitochondrial proteins.

Supplementary Figure 4

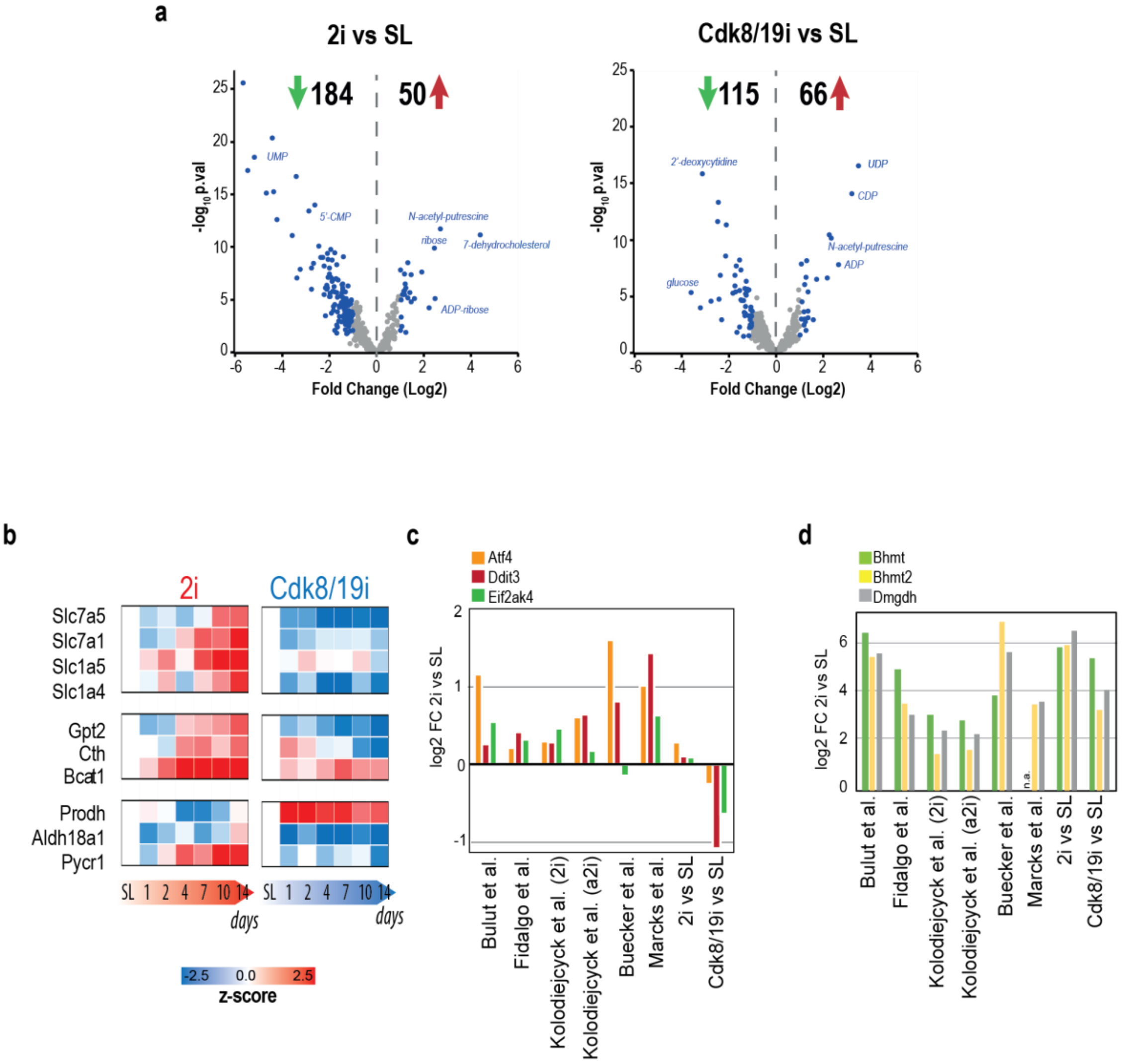

(A) Volcano plots showing the results from the differential expression analysis of the metabolomes of 2i vs S/L and Cdk8/19i vs S/L. In blue, statistically significant metabolites.

(B) Heatmap with the zscore normalized expression levels of Atf4 targets and proline biosynthesis proteins.

(C)Transcriptomic fold change (2i vs S/L) for Atf3, Ddit3 and Eif2aka4 from different published datasets: Lynch et al[9], Bulut-Karslioglu et al[10], Fidalgo et al[11], Kolodziejczyk et al[12], Buecker et al[17] and Marks et al[13].

(D)Transcriptomic fold change (2i vs S/L) for Bhmt, Bhmt2 and Dmgdh from different published datasets: Lynch et al[9], Bulut-Karslioglu et al[10], Fidalgo et al[11], Kolodziejczyk et al[12], Buecker et al[17] and Marks et al[13]. ‘na’ indicates that the data for that gene was not available in that dataset.

Supplementary Figure 5

a

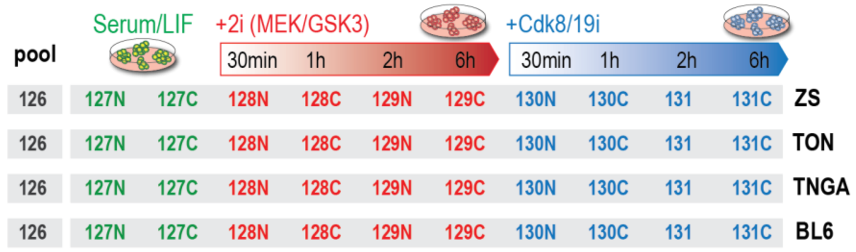

b

	Total MS/MS	MS/MS identified	Total peptides	Phospho-peptides	Phosphosites (STY)
TMT #1 - TON	1,005,999	161,179	20,409	18,904	22,266
TMT #2 -TNGA	1,016,291	165,906	19,805	18,364	21,133
TMT #3 - ZS	1,001,935	165,773	20,606	19,174	22,946
TMT #4 - BL6	1,011,427	157,574	19,767	18,685	22,314

c

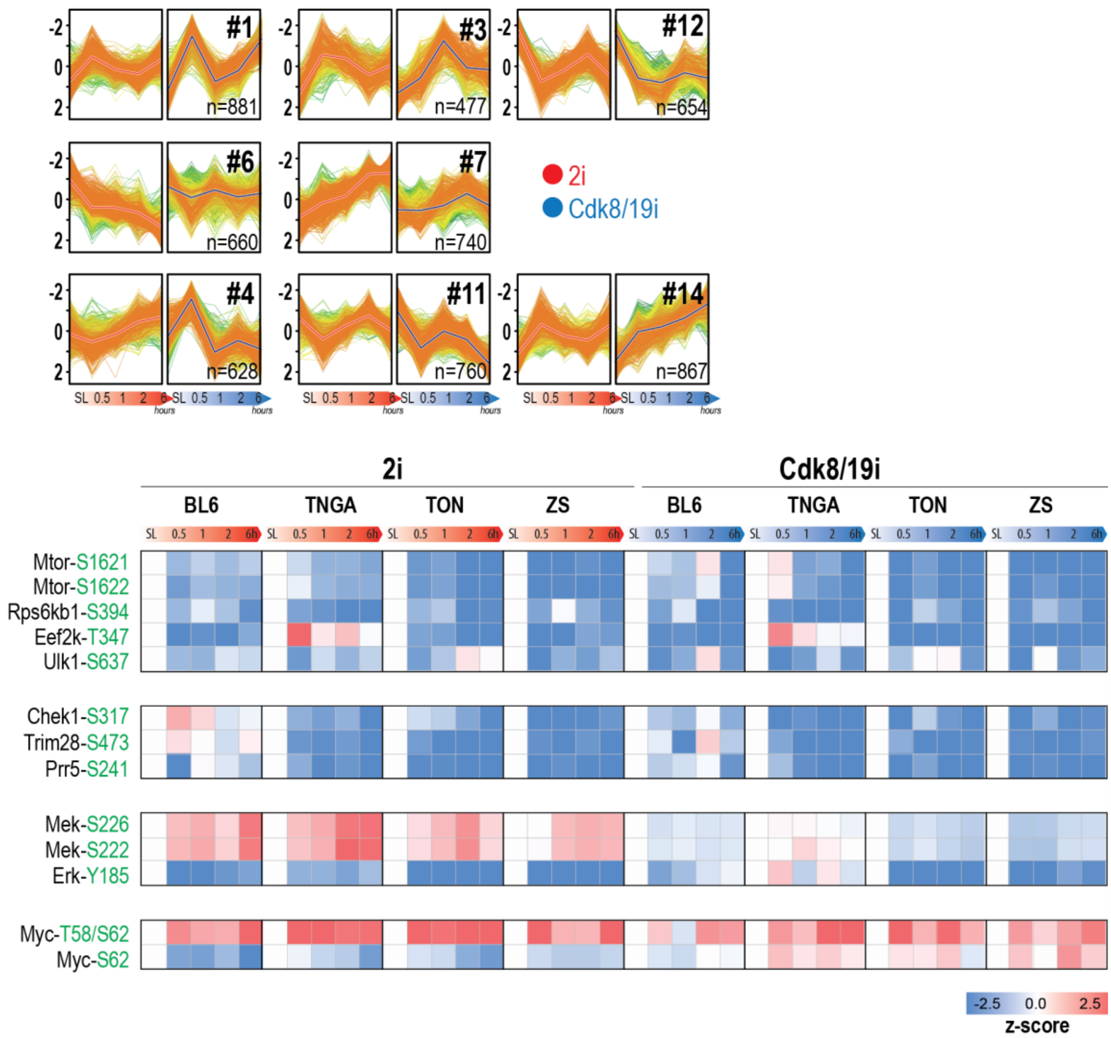

(A) Experimental design and labeling scheme for the phosphoproteome time course analysis of four mES cell lines.

(B) Summary of identifications for each independent TMT11plex experiment.

(C) Temporal clusters showing different kinetics in each treatment. Red and blue lines indicate the centroid of the distribution.

(D) Changes in phosphoryation (z-score normalized) in each of the four cell lines analyzed in this study for p-sites shown in Figure 6D-H.

Supplementary Figure 6

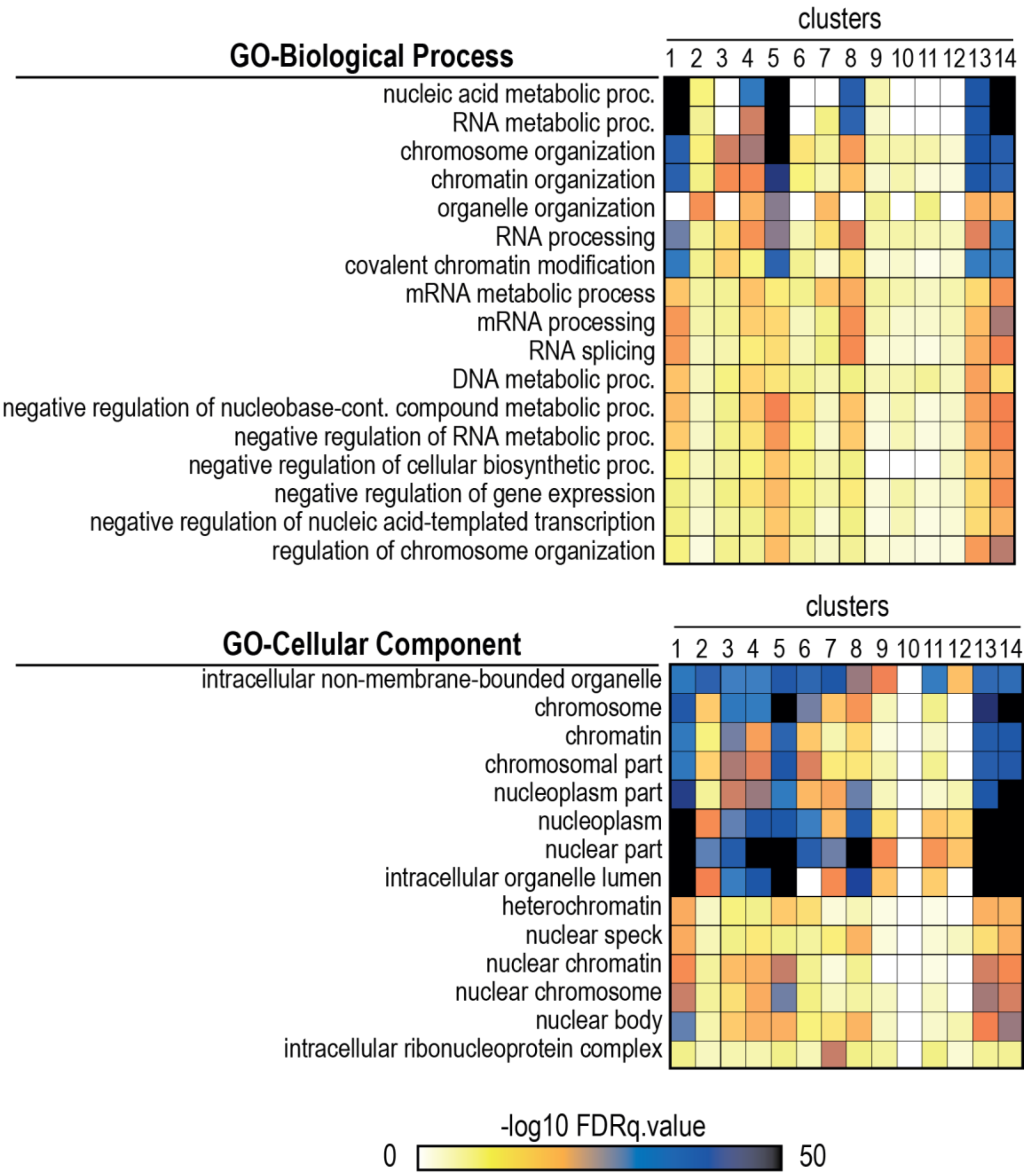

Gene Ontology (GO) enrichment analyses for phosphoproteins contained in the specified cluster number. The heatmap represents the significance level found for each term in the respective cluster using Biological Process (BP) and Cellular Component (CC).

Supplementary Figure 7

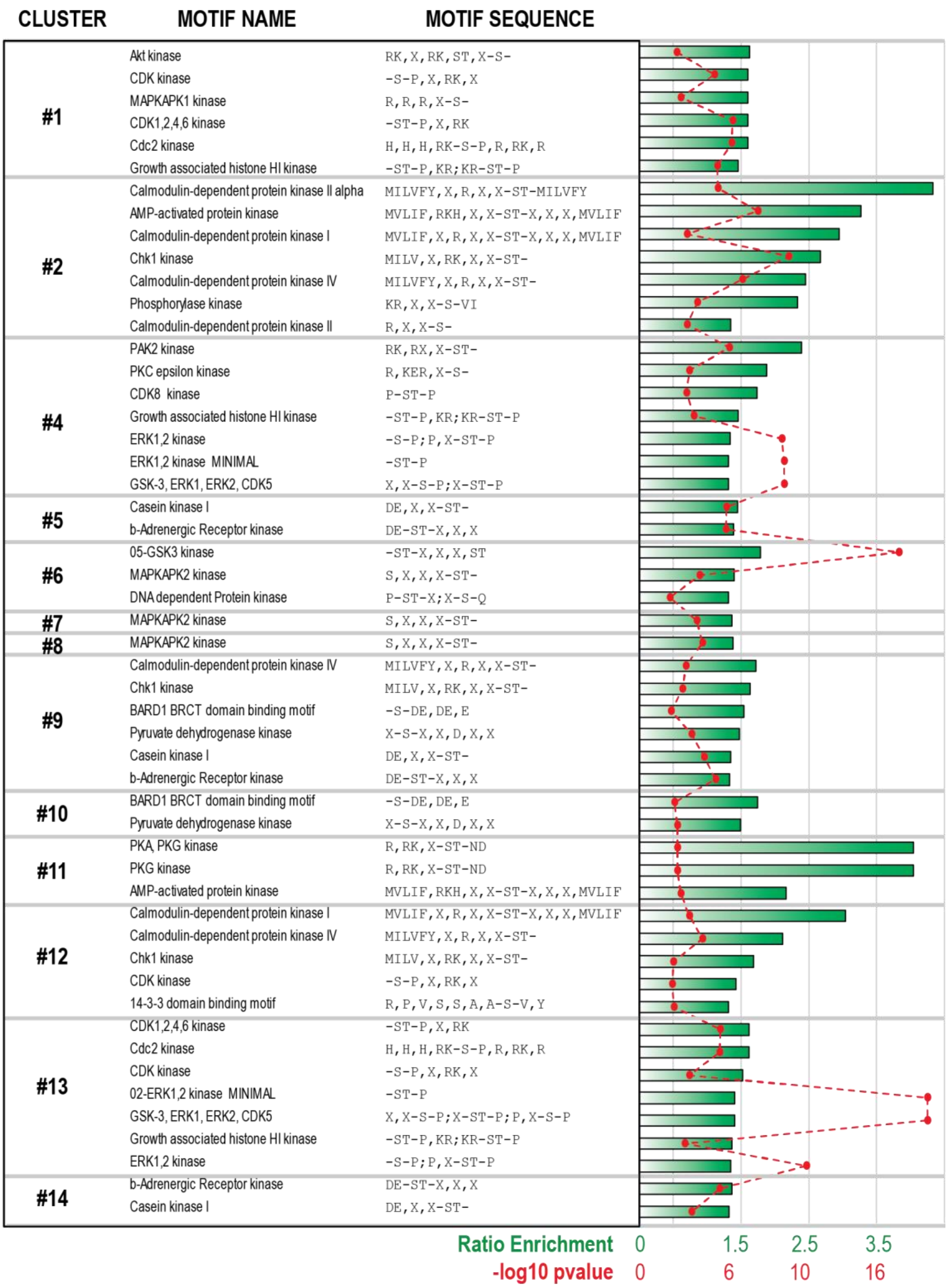

Phosphorylation motifs found statistically enriched (q-value<0.01, enrichment factor>1.3) in each phospho-cluster (N=4 independent cell lines). Linear motifs were extracted using Perseus and mapped to our data sets. A Fisher exact test (two-sided) was used to calculate the significance value in each case. Benjamini-Hochberg was used for multiple hypothesis testing correction. All the identified phosphosites in our study were used as background (N= 13,593 phospho-sites) and the corresponding enrichment ratios are shown.

Supplementary Figure 8

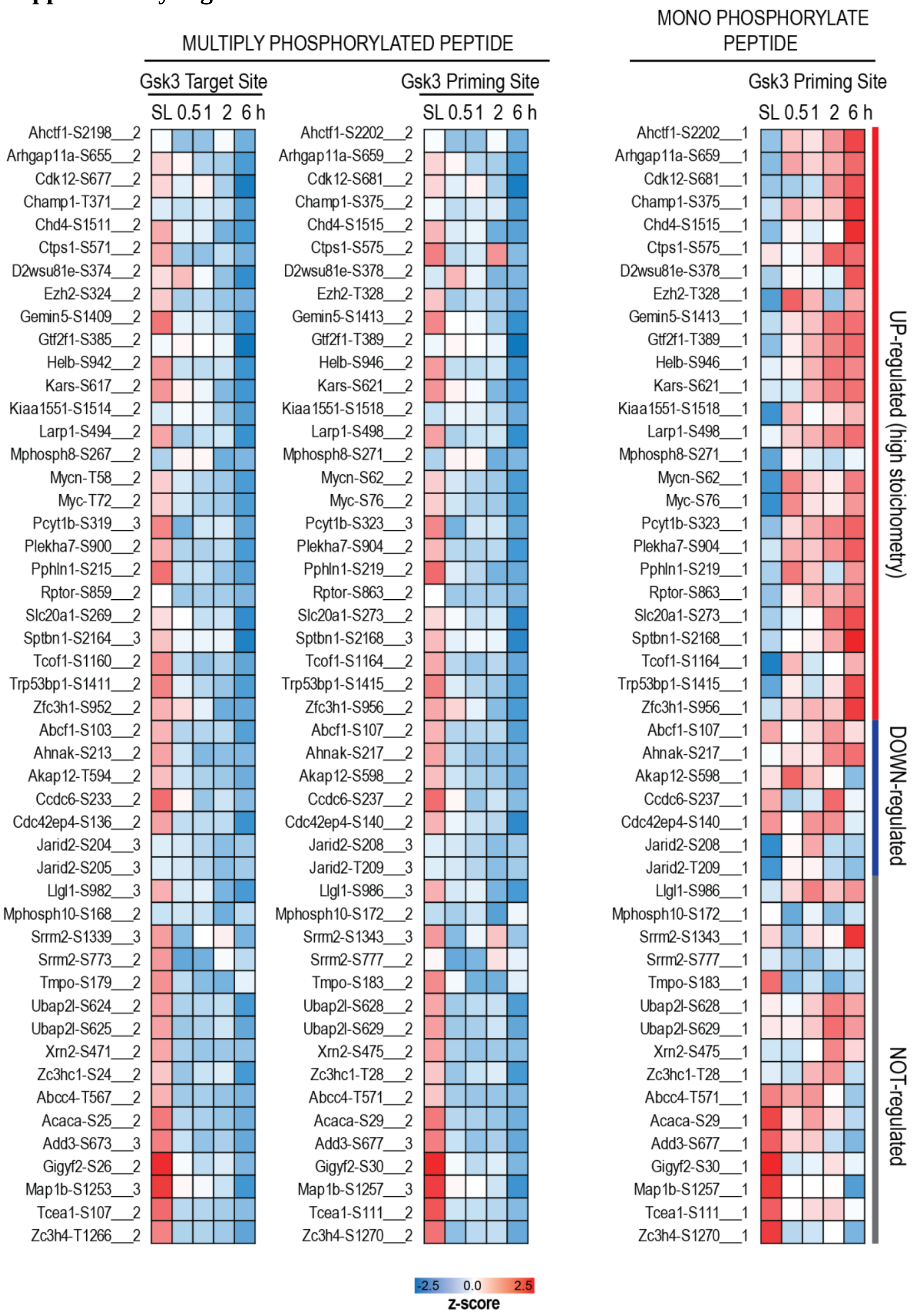

Potential GSK3 regulated sites after 2i treatment. Heatmap of phospho-sites that match the GSK3 motif S/T-XXX-S/T, together with the priming site for the doubly phosphorylated peptide, and the same site but for the mono-phosphorylated peptide.

Supplementary Figure 9

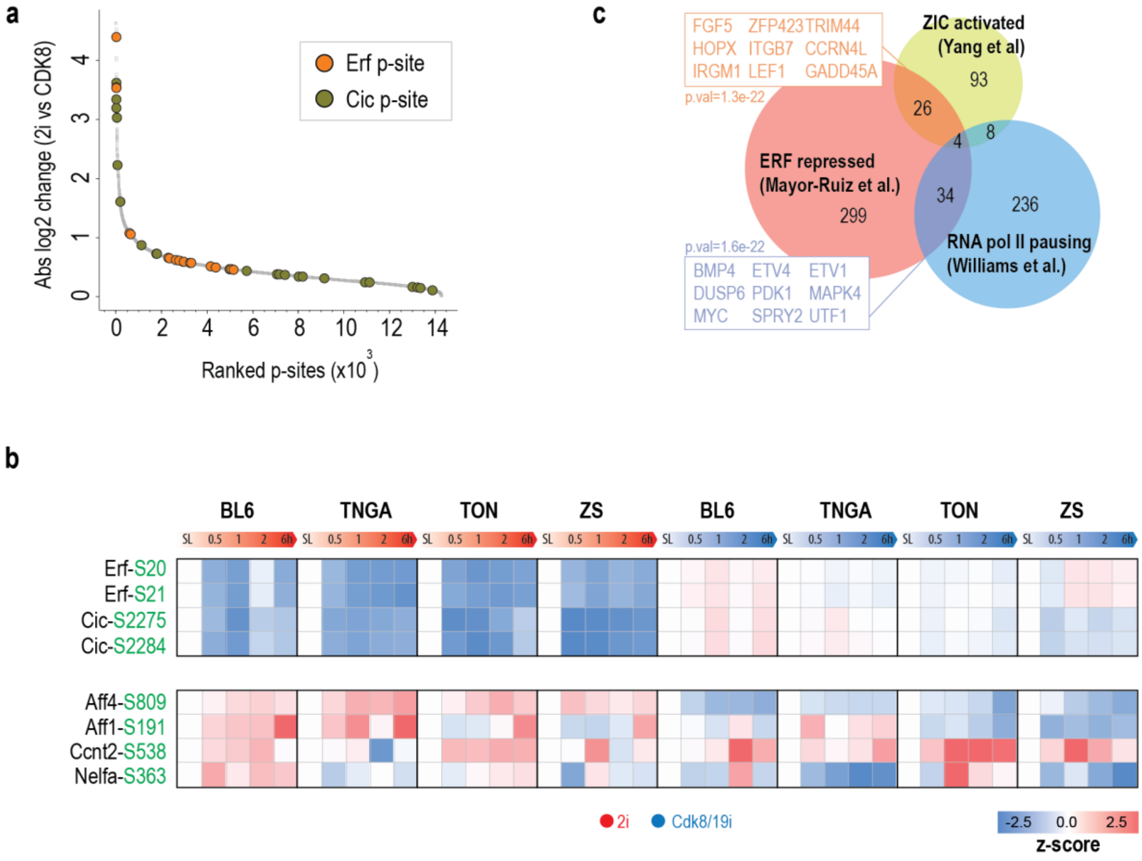

d

	Gene Set	SIZE	2i vs S/L ES	NES	NOM p-val	FDR q-val	FWER p-val	RANK AT MAX	Cdk8/19i vs S/L ES	NES	NOM p-val	FDR q-val	FWER p-val	RANK AT MAX
Protein (1 day)	CIC TARGETS	26	-0.475	-1.497	0.044	0.081	0.511	685	-0.243	-0.755	0.839	0.883	1.000	774
	ERF REPRESSOR	56	-0.702	-2.590	0.000	0.000	0.000	342	-0.420	-1.535	0.011	0.079	0.408	170
	ERF ACTIVATOR	46	0.556	2.041	0.000	0.001	0.002	589	-0.372	-1.290	0.131	0.246	0.932	548
	SUPER-ENHANCERS	67	0.308	1.201	0.159	0.207	0.982	231	0.494	1.902	0.000	0.007	0.009	571
Protein (2 day)	CIC TARGETS	26	-0.364	-1.133	0.299	0.462	0.997	555	-0.336	-1.044	0.375	0.540	1.000	775
	ERF REPRESSOR	56	-0.704	-2.571	0.000	0.000	0.000	394	-0.484	-1.775	0.000	0.022	0.044	599
	ERF ACTIVATOR	46	0.666	2.377	0.000	0.000	0.000	605	0.400	1.414	0.049	0.126	0.690	447
	SUPER-ENHANCERS	67	0.359	1.361	0.057	0.110	0.838	486	0.559	2.119	0.000	0.000	0.000	373
Protein (4 day)	CIC TARGETS	26	-0.404	-1.262	0.152	0.207	0.934	685	-0.270	-0.833	0.708	0.791	1.000	1058
	ERF REPRESSOR	56	-0.656	-2.403	0.000	0.000	0.000	347	-0.497	-1.817	0.002	0.012	0.031	613
	ERF ACTIVATOR	46	0.641	2.254	0.000	0.000	0.000	599	-0.352	-1.225	0.165	0.210	0.986	349
	SUPER-ENHANCERS	67	0.426	1.609	0.006	0.032	0.207	243	0.528	2.062	0.000	0.000	0.000	659
Protein (7 day)	CIC TARGETS	26	-0.429	-1.339	0.112	0.152	0.865	709	-0.466	-1.471	0.041	0.075	0.593	569
	ERF REPRESSOR	56	-0.662	-2.463	0.000	0.000	0.000	762	-0.567	-2.033	0.000	0.001	0.001	568
	ERF ACTIVATOR	46	0.578	2.088	0.000	0.000	0.000	670	-0.416	-1.444	0.039	0.082	0.679	444
	SUPER-ENHANCERS	67	0.365	1.419	0.026	0.179	0.646	160	0.475	1.952	0.000	0.008	0.010	479
Protein (10 day)	CIC TARGETS	26	-0.260	-0.784	0.789	0.899	1.000	288	-0.370	-1.141	0.289	0.357	0.998	666
	ERF REPRESSOR	56	-0.702	-2.515	0.000	0.000	0.000	479	-0.520	-1.922	0.002	0.006	0.007	585
	ERF ACTIVATOR	46	0.739	2.584	0.000	0.000	0.000	491	0.331	1.192	0.189	0.393	0.980	213
	SUPER-ENHANCERS	67	0.429	1.608	0.011	0.026	0.197	390	0.521	2.022	0.000	0.001	0.002	701
Protein (14 day)	CIC TARGETS	26	0.262	0.810	0.753	0.834	1.000	857	-0.409	-1.262	0.162	0.250	0.969	420
	ERF REPRESSOR	56	-0.643	-2.373	0.000	0.000	0.000	736	-0.511	-1.903	0.000	0.003	0.010	206
	ERF ACTIVATOR	46	0.688	2.466	0.000	0.000	0.000	869	-0.372	-1.319	0.093	0.219	0.907	294
	SUPER-ENHANCERS	67	0.439	1.675	0.004	0.034	0.136	437	0.425	1.682	0.000	0.043	0.108	265

(A) Ranked phosphosites by their absolute difference between 2i and Cdk8/19i phosphorylation levels. Erf (orange) and Cic (green) phosphorylation sites identified in our dataset are shown.

(B) Changes in phosphoryation (z-score normalized) in each of the four cell lines analyzed in this study for p-sites shown in Figure 7A and 7D.

(C) Venn-diagram of genes regulated at different levels (Erf repressed, RNA pol II pausing and Zic activated) retrieved from published datasets: Mayor-Ruiz et al[19], Williams et al[20] and Yang et al[21].

(D) Table with the GSEA results for the indicated datasets at different time points of treatment with 2i or Cdk8/19i. Statistical significance values were calculated using Kolmogorov-Smirnov statistics implemented in the GSEA algorithm. P-values were corrected for multiple testing.

Supplementary Notes

Supplementary Note 1: Cdk8/19i recapitulates the transcriptional changes of 2i in both serum and serum-free conditions

The undefined composition of serum results in cell metastability because conflicting signaling pathways are activated. In contrast, 2i alone is sufficient to drive many of the effects typical of the naive state, and that is why 2i treatment has been commonly employed to induce the naïve state of pluripotency in the absence of serum. Consistent with this, while it is a common practice to remove serum from media and expand mESCs in 2i/LIF-based formula, a number of reports on naïve pluripotency do not do this (for example Finley et al[22]).

In the present report, we preferred to keep serum in all our experiments in order to focus on analyzing the direct effects of Cdk8/19i on naive pluripotency, and dismiss potential signals triggered by removal of serum. In this way, the specific effects of each kinase inhibitor can be studied in isolation, without any putative contribution of serum removal on our cell system. Nevertheless, in our prior report by Lynch et al[9], we compared serum versus serum-free (KSR) conditions, showing that the typical morphology of mESCs and the homogenous expression of Nanog-GFP in 2i+LIF (serum-free, KSR) was recapitulated also in Cdk8/19i+LIF (serum-free, KSR) (Fig.1A and Extended Data Fig.1A in Lynch et al[9]). We also showed that the transcriptome of Cdk8/19i overlaps significantly with 2i conditions, in both serum-containing and serum-free media (Fig.4A, Extended Data Fig.3E,F and Supplementary Table 2 in Lynch et al[9]).

Here, we show that global mRNA changes induced by Cdk8/19i in the presence of serum are phenocopied when Cdk8/19i is supplemented in a serum-free based media (KSR) (Pearson=0.607). This result is very similar to the effects of 2i in the presence or absence of serum (Pearson=0.543) (Supplementary Fig. 10). Indeed, with respect to reference serum/LIF conditions, Cdk8/19i and 2i show a much better correlation in KSR-based media (Pearson=0.714) than in serum-containing media (Pearson=0.337), demonstrating that serum deprivation has an effect on its own and, consequently, to analyze the effects of Cdk8/19i in naive pluripotency, our experiments are better performed in the presence of serum. Moreover, some of the findings described in our work are also reproduced in serum-free conditions. For instance, we have seen that mitochondrial proteins are more up-regulated at the protein level than the mRNA level also in serum-free conditions (Supplementary Note 3), confirming that post-transcriptional regulation is prevalent in the naive pluripotent state, independently of serum.

Supplementary Figure 10

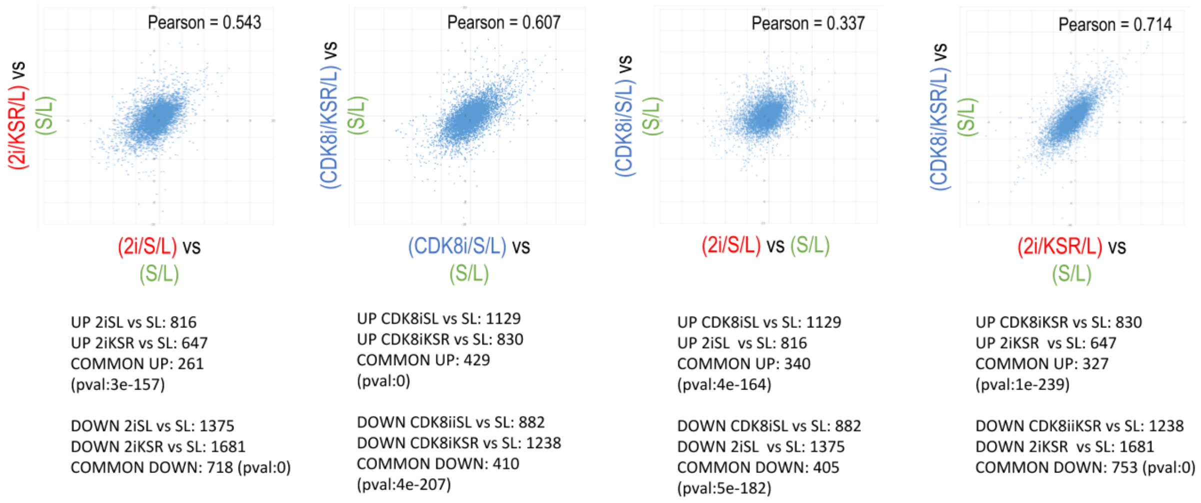

Correlation Plots between mRNA expression levels in mESCs grown in 2i or Cdk8/19i with Serum/LIF or in the absence of serum (KSR). Below, number of differentially expressed transcripts in each condition (two-sample t-test, FDR 5%, log2 fold change > 1), and the commonly regulated ones (p.value indicates the significance of the overlap based on a hypergeometric test). Source data are available as Source Data file.

Supplementary Note 2: Post-transcriptional regulation in naïve mESCs

In their report, Atlasi and colleagues focused on the translational landscape of naïve pluripotency. They show that 2i possesses a higher ribosome density on a set of mRNAs but, surprisingly, this is not reflected in concomitant protein changes, which is attributed by the authors to translational buffering. In addition, they show that proteome changes between pluripotent states are mainly driven by transcriptional rewiring. In light of one of our findings regarding the presence of post-transcriptional regulation in the naive state, Atlasi's results seem somehow discrepant with our findings. We have now examined the work from Atlasi et al[15]. and found evidence of post-transcriptional regulation in their data too, corroborating thereby our results and providing a potential mechanism, which integrates the key findings of both studies.

In the analyses of RNA, Ribosomal Foot-Prints (RFP) and proteins levels, Atlasi attributed protein changes as "transcription, translation or post-translation" on the basis of defined cut-offs (Fig. 3D). The established cut-offs for "protein changes" were stricter (Fold-Change > 3) than those used for transcriptional (RNA-seq) and translational (RFP) changes (Fold-Change > 2). These different criteria might impose a bias that favoured transcription as the main driver in defining the expression programs of pluripotent states. However, to better assess the contribution of each regulatory layer in gene expression control, we believe that smaller divergences between mRNA, RFP and protein levels should be accounted for. Indeed, using a 2-D enrichment approach that omits the need of arbitrary cut-offs, we have found notable differences between mRNA and RFP levels in Atlasi's data. This analysis reveals that genes regulated mainly at the translational level (RNA < RFP) are significantly enriched in endoplasmic reticulum and plasma membrane (FDR=0) (Supplementary Figure 11A-B). Importantly, these changes were only apparent after day 7, coincident with the down-regulation of Lin28 in Atlasi's time series (Supplementary Figure 11C). Therefore, these results support our findings, and others[23], on the role of LIN28 as a suppressor of ER-associated translation in pluripotency.

Noteworthy, the 2D-enrichment analysis of Atlasi's data did not show any differences in the translation of mitochondrial proteins. However, when comparing protein vs RNA levels, we found a significant difference, as mitochondrial components were more up-regulated at the protein level than they were at the mRNA level (p.val=2.8e-19) (Supplementary Figure 11D). Interestingly, this divergence is synchronized with the down-regulation of LIN28, in agreement with our hypothesis. In addition, we obtained very similar results when comparing RFP and protein levels (Supplementary Figure 11D). Therefore, Atlasi's data independently confirm our findings on the presence of post-transcriptional regulation of mitochondrial proteins in naïve cells.

An interesting question arises from these results concerning the true contribution of translation (as measured by RFP) to the final protein concentration levels (as measured by MS). Given that RFP is a direct reflection of translational activity, it is intriguing that the up-regulation of mitochondrial protein levels (MS) is not detected in the RFP of Atlasi and colleagues. Below, we propose several explanations (or a combination of them) which might explain this result:

- The naïve state may increase the stability of mitochondrial proteins (for instance, by means of post-translational modifications). However, the apparent role of LIN28 degradation in the up-regulation of these proteins argues against this hypothesis.

- Under specific circumstances such as stress, cells undergo global changes in transcription or translation rates. Accounting incorrectly for these changes can distort gene-specific measurements. In this scenario, pulsed-SILAC is reported to detect changes in protein

synthesis more accurately than RFP[24]. Given that 2i exhibits lower transcription and translation rates than S/L[10], a result confirmed in our data (Fig 3F), it may be conceivable that normalization of RFP in Atlasi's data might lead to inaccurate estimates of translation efficiencies as seen before by others[25] and might mask actual changes in the footprints of mitochondrial proteins.

● RFP is normally measured in bulk cell populations, providing an average estimate of translational activity. However, translation is often spatially regulated in organelles[26]. In fact, proximity-specific ribosome profiling has revealed pervasive co-translational targeting to ER[27] and mitochondria[28]. Therefore, a local increase in translation at the surface of these organelles might be unnoticed when using a whole cell RFP strategy like in Atlasi.

● Translationally inactive mRNAs can be engaged into ribonucleoproteins and stalled/paused in polysomes due to physiological surveillance, stress and regulatory mechanisms[29]. While inactive ribosomes unbound to transcripts do not present a problem, Ribo-seq does not differentiate actual protected footprints of translating polysomes from RNA fragments sequestered in inactive/stalled ribosomes, leading to possible misinterpretations of translation occupancies[30]. In S/L mESCs, ribosomes in ER and mitochondria are pre-loaded with their target mRNAs but, because of LIN28 binding, do not undergo active translation. Since the RFP approach employed by Atlasi cannot distinguish inactive from active ribosomes, the overall net change for mitochondrial mRNAs footprints upon LIN28 degradation in 2i might therefore remain constant. Indeed, translational control of pre-existing mRNAs allows faster protein synthesis than the transcription of new mRNAs, which may explain the prominence of translational regulation in stress responses[31,32].

Supplementary Figure 11

a

GO Term	Day 1 (2i vs SL)				Day 3 (2i vs SL)				Day 7 (2i vs SL)				Long-Term (2i vs SL)			
	RNA	RFP	P.val	FDR	RNA	RFP	P.val	FDR	RNA	RFP	P.val	FDR	RNA	RFP	P.val	FDR
integral to membrane	-0.508	-0.502	0	0	-0.313	-0.126	0	0	-0.275	0.053	0	0	-0.069	0.106	0	0
intrinsic to membrane	-0.511	-0.505	0	0	-0.318	-0.129	0	0	-0.274	0.056	0	0	-0.070	0.107	0	0
endoplasmic reticulum part	-0.473	-0.448	0	0	-0.308	-0.096	0	0	-0.292	-0.034	0	0	-0.086	0.111	0	0
membrane part	-0.405	-0.394	0	0	-0.249	-0.092	0	0	-0.216	0.049	0	0	-0.051	0.087	0	0
ribonucleoprotein complex	0.159	0.295	2.22E-16	2.46E-14	0.190	0.144	6.51E-07	3.28E-05	0.048	-0.209	0	0	-0.205	-0.085	6.04E-11	1.34E-08
nucleobase-containing compound metabolic process	0.125	0.140	6.64E-12	1.46E-08	0.096	0.022	4.26E-09	5.34E-06	0.037	-0.118	0	0	-0.028	-0.096	4.41E-10	1.94E-06
membrane	-0.190	-0.191	0	0	-0.124	-0.030	1.07E-14	1.97E-12	-0.088	0.073	0	0	0.013	0.071	2.44E-07	1.81E-05
cellular nitrogen compound metabolic process	0.116	0.132	3.10E-11	5.44E-08	0.100	0.022	3.44E-10	6.04E-07	0.039	-0.111	0	0	-0.020	-0.083	6.86E-09	2.01E-05
RNA binding	0.090	0.191	2.26E-10	6.77E-08	NaN	NaN	NaN	NaN	-0.022	-0.255	0	0	-0.177	-0.167	5.43E-08	2.09E-05

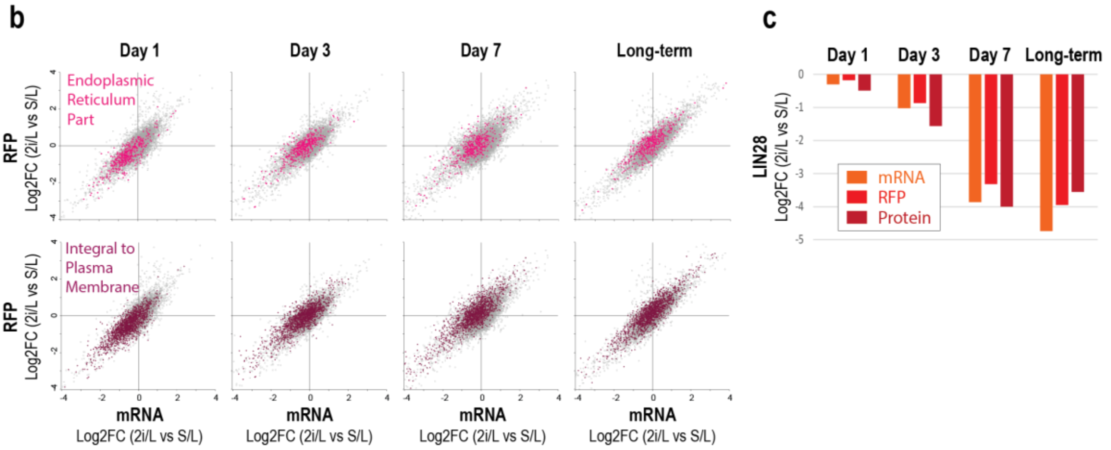

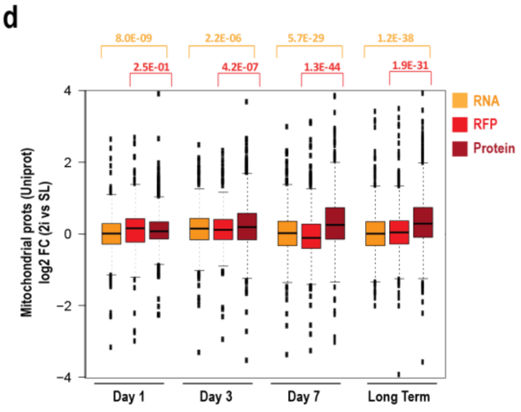

(A) 2D enrichment analysis of mRNA and RFP (Ribosome Footprint Profiling) data from Atlasi et al[15] reveals multiple GO terms significantly enriched. Terms are sorted by the FDR in the long-term condition. Only the top terms are shown in the figure. Statistical significance was calculated using two-sided two-sample test for difference of means with FDR-BH correction, implemented in the 2D enrichment analysis in Perseus.

(B) Scatterplots of mRNA and RFP data at the indicated time-points, highlighting genes annotated as GOCC-Endoplasmic Reticulum Part and GOCC-Integral to plasma membrane.

(C) mRNA, RFP and protein log2 fold change (2i vs S/L) for Lin28 gene across the time-points analyzed by Atlasi et al[15].

(D) Log2 ratios (2i/L vs S/L) of mitochondrial proteins (as in UniprotKB) extracted from mRNA, RFP and protein (MS) measurements reveal pervasive up-regulation at the protein level compared to mRNA and RFP levels. Boxplot represent values for N=608

mitochondrial proteins. For boxplot representation, center lines show the medians; box limits indicate the 25th and 75th percentiles as determined by R software; whiskers extend 1.5 times the interquartile range from the 25th and 75th percentiles, outliers are represented by dots. Statistical significance was calculated using a paired two-sample t-test for means, two sided. Source data are available as Source Data file.

Supplementary Note 3: Early molecular events upon 2i or Cdk8/19i treatment are mainly driven by changes in phosphorylation rather than protein abundance levels

Even at very early time points, such as the ones analyzed in the current study, the potential contribution of changes in protein abundance in the measured phospho-sites cannot be completely ruled-out. However, there are two major points suggesting that this important issue is minimized in our data:

(i) Phosphorylation changes were sampled at the very early moments of adding the 2i or Cdk8/19i (0.5, 1, 2 and 6 hours). This should enable us to capture the major signal transduction events driven by phosphorylation before gene expression changes take place. In support of this hypothesis, Yang et al[33] performed a multi-omic profiling of naive mESCs exiting pluripotency and showed that widespread proteome changes were only evident after 12h. They demonstrated that phosphorylation cascades precede ordered waves of epigenomic, transcriptomic, and proteomic changes (Fig 2D in Yang et al[33]). Although this was found in the reverse process (the transition of naive towards "primed EpiSC"), a similar behavior of molecular events could be expected in our system ("primed S/L" to naive). In line with this, we have compared overall phosphorylation changes with protein levels at day 1 and did not find any correlation (Supplementary Fig. 12A). We found similar results in Yang's data for matched phosphorylation-proteome data at 30 and 60 min (Supplementary Fig. 12B). In agreement with Yang et al[33] (Fig 2E), we also found that, in both 2i and Cdk8/19i, the magnitude of phosphorylation changes in the first 6h is superior to that of protein changes at day 1 (Supplementary Fig. 12C-D). Moreover, proteomic changes at day 1 are significantly minor and notably smaller compared to subsequent time points (2, 4, 7, 10 and 14 days), suggesting that in the first 6h of 2i and Cdk8/19i, regulatory events are mainly driven by changes in phosphorylation rather than protein abundance.

(ii) Molecular information can often be transmitted in a site-specific manner: different sites in the same protein have different functions and, therefore, can be subject to differential regulation. Consequently, if changes in protein abundance are the major determinants in the regulation of its function (at a given time/stimuli), it might be expected that all the identified phosphopeptides for this protein should exhibit nearly identical kinetics. However, we found in our data a very large number of proteins that showed site-specific regulation of their p-sites (Supplementary Figure 12E). Nevertheless, functional cooperativity within proximal phosphorylation sites is also a prevalent regulatory mechanism of proteins. This might also explain the nearly identical kinetics observed for certain p-sites, (S1621 and S1622 in mTOR. Fig 6D).

Supplementary Figure 12

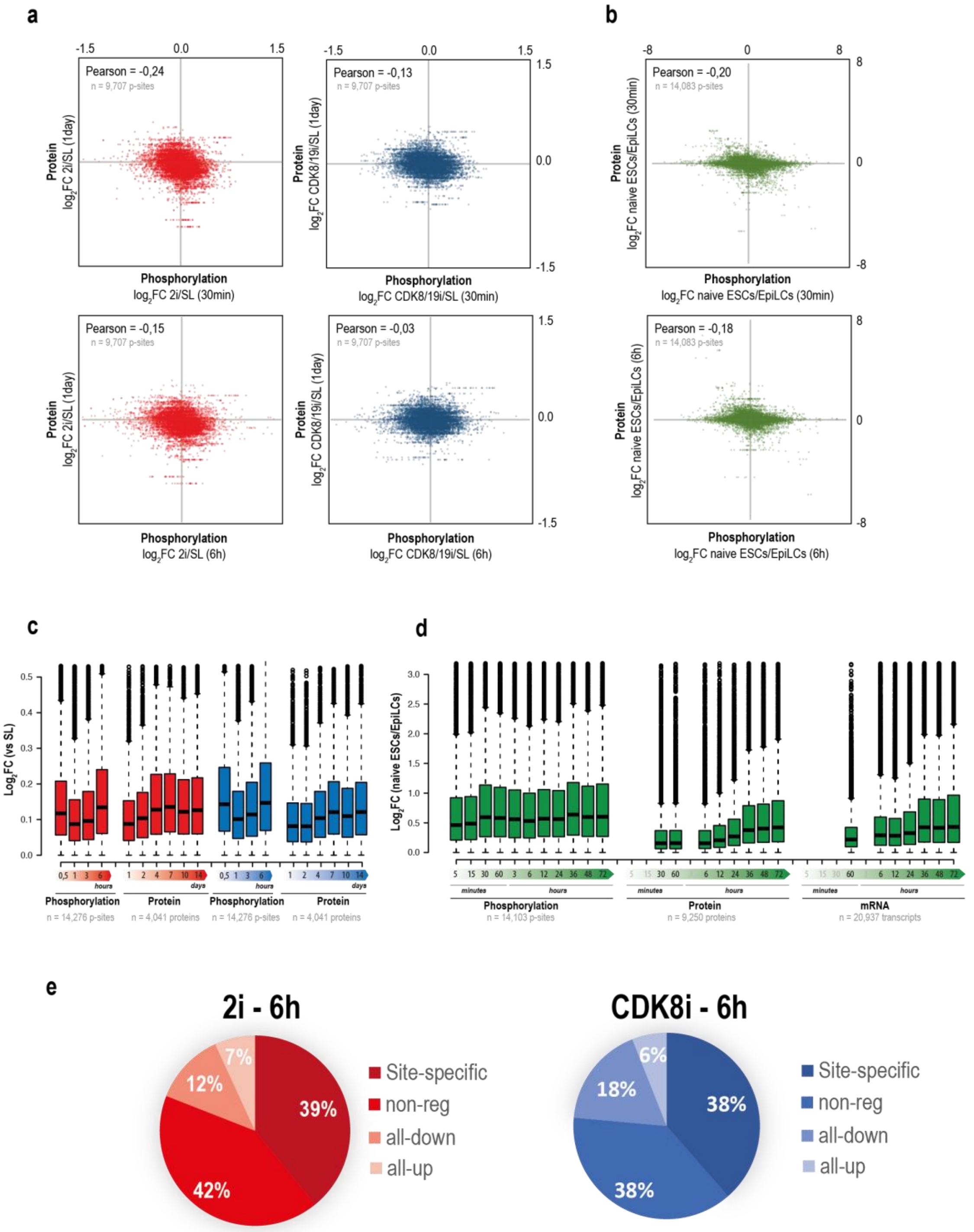

(A) Correlation plot between protein level at 1day of treatment versus phospho-site level at 30 minutes and 6 hours of treatment for 2i (red) and Cdk8/19i (blue) conditions. Correlation is calculated by a Pearson's correlation test, two-tailed. The size (n = number of data points) for each plot is indicated in grey.

(B) Correlation plot between protein level at 1day of treatment versus phospho-site level at 30 minutes and 6 hours of treatment for 2i for data obtained from Yang et al[33]. The size (n = number of data points) for each plot is indicated in grey.

(C) Boxplots of absolute fold-change in phospho-site and protein level at all time points evaluated for 2i or Cdk8/19i treatment. Center lines in boxpots show the medians; box limits indicate the 25th and 75th percentiles as determined by R software; whiskers extend 1.5 times the interquartile range from the 25th and 75th percentiles, outliers are represented by dots. The size (n = number of data points) for each plot is indicated in grey. Source data are available as Source Data file.

(D) Boxplots of absolute fold-change in phospho-site, protein and mRNA level at all time points evaluated for the naïve to primed transition in Yang et al[33]. The size (n = number of data points) for each plot is indicated in grey. Source data are available as Source Data file.

(E) Pie charts showing the percentage of proteins for which the identified phospho-sites are regulated in a site-specific manner or all sites within a protein regulated in the same direction or not regulated at 6 hours of 2i or Cdk8/19i treatment.

Supplementary References

1. Wiśniewski, J. R. *et al.* Universal sample preparation method for proteome analysis. *Nat. Methods* **6**, 359–62 (2009).

2. Cox, J. & Mann, M. MaxQuant enables high peptide identification rates, individualized p.p.b.-range mass accuracies and proteome-wide protein quantification. *Nat. Biotechnol.* **26**, 1367–72 (2008).

3. Cox, J. *et al.* Andromeda: a peptide search engine integrated into the MaxQuant environment. *J. Proteome Res.* **10**, 1794–805 (2011).

4. Ritchie, M. E. *et al.* limma powers differential expression analyses for RNA-sequencing and microarray studies. *Nucleic Acids Res.* **43**, e47–e47 (2015).

5. Leek, J. T., Johnson, W. E., Parker, H. S., Jaffe, A. E. & Storey, J. D. The sva package for removing batch effects and other unwanted variation in high-throughput experiments. *Bioinformatics* **28**, 882–3 (2012).

6. Wieczorek, S. *et al.* DAPAR & ProStaR: software to perform statistical analyses in quantitative discovery proteomics. *Bioinformatics* **33**, 135–136 (2017).

7. HaileMariam, M. *et al.* S-Trap, an Ultrafast Sample-Preparation Approach for Shotgun Proteomics. *J. Proteome Res.* **17**, 2917–2924 (2018).

8. Bindea, G. *et al.* ClueGO: a Cytoscape plug-in to decipher functionally grouped gene ontology and pathway annotation networks. *Bioinformatics* **25**, 1091–1093 (2009).

9. Lynch, C. J. *et al.* Global hyperactivation of enhancers stabilizes human and mouse naive pluripotency through inhibition of CDK8/19 Mediator kinases. *Nat. Cell Biol.* **22**, 1223–1238 (2020).

10. Bulut-Karslioglu, A. *et al.* Inhibition of mTOR induces a paused pluripotent state. *Nature* **540**, 119–123 (2016).

11. Fidalgo, M. *et al.* Zfp281 Coordinates Opposing Functions of Tet1 and Tet2 in Pluripotent States. *Cell Stem Cell* **19**, 355–369 (2016).

12. Kolodziejczyk, A. A. *et al.* Single Cell RNA-Sequencing of Pluripotent States Unlocks Modular Transcriptional Variation. *Cell Stem Cell* **17**, 471–485 (2015).

13. Marks, H. *et al.* The Transcriptional and Epigenomic Foundations of Ground State Pluripotency. *Cell* **149**, 590–604 (2012).

14. Boroviak, T. *et al.* Lineage-Specific Profiling Delineates the Emergence and Progression of Naive Pluripotency in Mammalian Embryogenesis. *Dev. Cell* **35**, 366–382 (2015).

15. Atlasi, Y. *et al.* The translational landscape of ground state pluripotency. *Nat. Commun.* **11**, 1–13 (2020).

16. Rafiee, M.-R., Girardot, C., Sigismondo, G. & Krijgsveld, J. Expanding the Circuitry of Pluripotency by Selective Isolation of Chromatin-Associated Proteins. *Mol. Cell* **64**, 624–635 (2016).

17. Buecker, C. *et al.* Reorganization of enhancer patterns in transition from naive to primed pluripotency. *Cell Stem Cell* **14**, 838–53 (2014).

18. Hafner, M. *et al.* Identification of mRNAs bound and regulated by human LIN28 proteins

and molecular requirements for RNA recognition. *RNA* **19**, 613–626 (2013).

19. Mayor-Ruiz, C. *et al.* ERF deletion rescues RAS deficiency in mouse embryonic stem cells. *Genes Dev.* **32**, 568–576 (2018).

20. Williams, L. H. *et al.* Pausing of RNA Polymerase II Regulates Mammalian Developmental Potential through Control of Signaling Networks. *Mol. Cell* **58**, 311–322 (2015).

21. Yang, S.-H. *et al.* ZIC3 Controls the Transition from Naive to Primed Pluripotency Article ZIC3 Controls the Transition from Naive to Primed Pluripotency. *Cell Rep.* **27**, 3215–3227 (2019).

22. Finley, L. W. S. *et al.* Pluripotency transcription factors and Tet1/2 maintain Brd4-independent stem cell identity. *Nat. Cell Biol.* **20**, 565–574 (2018).

23. Cho, J. *et al.* LIN28A Is a Suppressor of ER-Associated Translation in Embryonic Stem Cells. *Cell* **151**, 765–777 (2012).

24. Liu, T. Y. *et al.* Time-Resolved Proteomics Extends Ribosome Profiling-Based Measurements of Protein Synthesis Dynamics. *Cell Syst.* **4**, 636-644.e9 (2017).

25. Ingolia, N. T. Tracking the Missing Footprints of Idle Ribosomes. *Cell Systems* vol. 4 583–584 (2017).

26. Fazal, F. M. *et al.* Atlas of Subcellular RNA Localization Revealed by APEX-Seq. *Cell* **178**, 473-490.e26 (2019).

27. Jan, C. H., Williams, C. C. & Weissman, J. S. Principles of ER cotranslational translocation revealed by proximity-specific ribosome profiling. *Science (80-.).* **346**, (2014).

28. Williams, C. C., Jan, C. H. & Weissman, J. S. Targeting and plasticity of mitochondrial proteins revealed by proximity-specific ribosome profiling. *Science (80-.).* **346**, 748–751 (2014).

29. Yordanova, M. M. *et al.* AMD1 mRNA employs ribosome stalling as a mechanism for molecular memory formation. *Nature* **553**, 356–360 (2018).

30. Clamer, M. *et al.* Active Ribosome Profiling with RiboLace. *Cell Rep.* **25**, 1097-1108.e5 (2018).

31. Spriggs, K. A., Bushell, M. & Willis, A. E. Translational Regulation of Gene Expression during Conditions of Cell Stress. *Molecular Cell* vol. 40 228–237 (2010).

32. Ingolia, N. T. Ribosome Footprint Profiling of Translation throughout the Genome. *Cell* vol. 165 22–33 (2016).

33. Yang, P. *et al.* Multi-omic Profiling Reveals Dynamics of the Phased Progression of Pluripotency. *Cell Syst.* **8**, 427-445.e10 (2019).
