# Supplementary material for: Dissection of two routes to naïve pluripotency using different kinase inhibitors
Source: Nat Commun. 2021 Mar 25;12:1863. doi: 10.1038/s41467-021-22181-5 (PMC7994667; doi:10.1038/s41467-021-22181-5)
Supplement: Supplementary file 3 — Description of Additional Supplementary Files [file 41467_2021_22181_MOESM3_ESM.docx]

File name: Supplementary Data 1

Description: Differential expression (log2 fold change and limma p-value) of the time course full proteome experiment and clustering. (Sheet 1.1.) Differential analysis results of the time course treatment with 2i or Cdk8/19i. Fold change (2i vs Serum/LIF and Cdk8/19i vs Serum/LIF) in log2, together with a categorical column (UP/DOWN) that indicates whether the value is significant in limma (two-sided moderated t-test, FC >0.1 or FC<-0.1 and FDR 5% Benjamini Hochberg) for the 4,408 proteins quantified in the long term time course experiment (7 time points during 2 weeks of treatment). Cluster column indicates to which cluster each protein has been assigned to. (Sheet 1.2.) Log2 transformed intensities from each different itraq8plex experiment. Raw data was normalized using cyclicloess for intra-experiment bias removal, and ComBat for inter-experiment batch effect removal.

File name: Supplementary Data 2

Description: Gene Ontology (Biological Process and Cellular Component) enrichment results obtained from ClueGO for each one of the clusters defined for the proteome (each cluster enrichment is shown in a separate sheet). Enrichment tests to calculate statistical significance for over-representation (one-sided) were based on a hypergeometric distribution with FDR correction by Benjamini-Hochberg.

File Name: Supplementary Data 3

Description: (Sheet 3.1) RNA and Protein correlation based on ratios of 2i vs Serum/LIF and Cdk8/19i vs Serum/LIF. RNA data was obtained from Lynch et al (PMID: 32989249) and protein values correspond to the last time point used in the proteome time course (14 days). (Sheet 3.2-3.5) Gene Ontology enrichment analysis (BP and CC) for proteins that show increased translation due to 2i (sheet 3.2-3.3) or Cdk8/19i (sheet 3.4-3.5) treatments. GO enrichment was performed in StringDB, statistical significance was calculated from an enrichment test, using FDR<5%.

File Name: Supplementary Data 4

Description: (Sheet 4.1) Differential expression (log2 fold change and p-value, limma two-sided moderated t-test) of the metabolomics experiment. (Sheet 4.2) Log2 transformed intensities from each different metabolomic sample (log2). Colored cells correspond to imputed values: blue means that imputation was perfomed using the "slsa" method in Prostar and orange indicates that the values were missing in an entire experimental condition and therefore imputed using "detquant" function in Prostar.

File Name: Supplementary Data 5

Description: (Sheet 5.1) Differential analysis results of the time course treatment with 2i or Cdk8/19i. Fold change (2i vs Serum/LIF and Cdk8/19i vs Serum/LIF) in log2, together with a categorical column (UP/DOWN) that indicates whether the value is significant in limma (two-sided moderated t-test, FC >0.1 or FC<-0.1 and FDR 5% Benjamini Hochberg) for the phosphosites quantified in the short term time course experiment (4 time points). (Sheet 5.2) Log2 transformed intensities from each different tmt11plex experiment. Raw data was normalized using cyclicloess for intra-experiment bias removal, and ComBat for inter-experiment batch effect removal.

File Name: Supplementary Data 6

Description: Gene Ontology (Biological Process and Cellular Component) enrichment q-values obtained from ClueGO for each one of the clusters defined for the temporal phospho-proteome. Enrichment tests to calculate statistical significance for over-representation (one-sided) were based on a hypergeometric distribution with FDR correction by Benjamini-Hochberg. Related to Supplementary figure 6.

File Name: Supplementary Data 7

Description: Phospho-proteome kinase motif analysis and fisher’s exact test for motif enrichment in each cluster defined for the phospho-proteome. Statistical significance was calculated by a Fisher exact test (two-sided, FDR<10%). Related to Supplementary figure 7.

File Name: Supplementary Data 8

Description: GSK3 motif analysis. Phospho-sites that show GSK3 phosphorylation motif in a doubly phosphorylated peptide, as well as their primed mon-phosphorylated counterpart. Phospho-site intensity is represented as a normalized z-score. Related to Supplementary figure 8.

File Name: Supplementary Data 9

Description: Reprocessed analysis results from RNASeq experiment in Lynch et al (PMID: 32989249). Differential expression between treatments (2i, CDK8/19i and CDK8/19-3i vs Serum/LIF) was performed using limma (two-sided moderated t-test) for which the p-values and FDR q-values are shown.
