# Supplementary material for: Dissection of two routes to naïve pluripotency using different kinase inhibitors
Source: Nat Commun. 2021 Mar 25;12:1863. doi: 10.1038/s41467-021-22181-5 (PMC7994667; doi:10.1038/s41467-021-22181-5)
Supplement: Supplementary file 13 — Reporting Summary [file 41467_2021_22181_MOESM13_ESM.pdf]

nature research

Corresponding author(s): Javier Muñoz

Last updated by author(s): Feb 11, 2021

Reporting Summary

Nature Research wishes to improve the reproducibility of the work that we publish. This form provides structure for consistency and transparency in reporting. For further information on Nature Research policies, see our Editorial Policies and the Editorial Policy Checklist.

Statistics

For all statistical analyses, confirm that the following items are present in the figure legend, table legend, main text, or Methods section.

n/a	Confirmed	
☐	☒	The exact sample size (n) for each experimental group/condition, given as a discrete number and unit of measurement
☐	☒	A statement on whether measurements were taken from distinct samples or whether the same sample was measured repeatedly
☐	☒	The statistical test(s) used AND whether they are one- or two-sided<br>*Only common tests should be described solely by name; describe more complex techniques in the Methods section.*
☒	☐	A description of all covariates tested
☒	☐	A description of any assumptions or corrections, such as tests of normality and adjustment for multiple comparisons
☐	☒	A full description of the statistical parameters including central tendency (e.g. means) or other basic estimates (e.g. regression coefficient) AND variation (e.g. standard deviation) or associated estimates of uncertainty (e.g. confidence intervals)
☐	☒	For null hypothesis testing, the test statistic (e.g. F, t, r) with confidence intervals, effect sizes, degrees of freedom and P value noted<br>*Give P values as exact values whenever suitable.*
☒	☐	For Bayesian analysis, information on the choice of priors and Markov chain Monte Carlo settings
☒	☐	For hierarchical and complex designs, identification of the appropriate level for tests and full reporting of outcomes
☐	☒	Estimates of effect sizes (e.g. Cohen's d, Pearson's r), indicating how they were calculated

Our web collection on statistics for biologists contains articles on many of the points above.

Software and code

Policy information about availability of computer code

Data collection	Data Analysis (Bruker) for proteomic data. Xcalibur (Q-Exactive software Tune version 2.9, Thermo) for phosphoproteomic data.
Data analysis	Proteomics data was analysed using MaxQuant software (versions 1.5.3.30 and 1.6.0.16), and processed using Perseus (v1.6.2.2) as well as with R (version 3.5). The following R packageswere used: sva (3.30.1), limma (3.36.1), cluster (2.0.7), multiClust (v1.14.12), Prostar (v1.12)). Differential analysis was done with limma function implemented within Prostar.

For manuscripts utilizing custom algorithms or software that are central to the research but not yet described in published literature, software must be made available to editors and reviewers. We strongly encourage code deposition in a community repository (e.g. GitHub). See the Nature Research guidelines for submitting code & software for further information.

Data

Policy information about availability of data

All manuscripts must include a data availability statement. This statement should provide the following information, where applicable:

- Accession codes, unique identifiers, or web links for publicly available datasets
- A list of figures that have associated raw data
- A description of any restrictions on data availability

The mass spectrometry proteomics data have been deposited to the ProteomeXchange Consortium via the PRIDE partner repository with the dataset identifier PXD018694 [http://proteomecentral.proteomexchange.org/cgi/GetDataset?ID=PXD018694]. The mass spectrometry metabolomics data have been deposited to the Metabolights database with the identifier MTBLS301 [https://www.ebi.ac.uk/metabolights/MTBLS301/descriptors].
Processed data from the following selected publications was used in the current publication: Bulut-Karslioglu et al16 (GSE81285, [https://www.ncbi.nlm.nih.gov/geo/query/acc.cgi?acc=GSE81285]), Marks et al5 (GSE23943, [https://www.ncbi.nlm.nih.gov/geo/query/acc.cgi?acc=GSE23943]), Buecker et al60 (GSE56138, [https://www.ncbi.nlm.nih.gov/geo/query/acc.cgi?acc=GSE56138]), Kolodziejczyk et al76 (E-MTAB-2600, [https://www.ebi.ac.uk/arrayexpress/experiments/E-

MTAB-2600/]), Fidalgo et al77 (GSE81045, [https://www.ncbi.nlm.nih.gov/geo/query/acc.cgi?acc=GSE81045]) and Lynch et al3 (GSE112208, [https://www.ncbi.nlm.nih.gov/geo/query/acc.cgi?acc=GSE112208].
Mice (Mus musculus) protein database was obtained from UniprotKB (Swiss-Prot and TrEMBL, https://www.uniprot.org/).

Field-specific reporting

Please select the one below that is the best fit for your research. If you are not sure, read the appropriate sections before making your selection.

☒ Life sciences ☐ Behavioural & social sciences ☐ Ecological, evolutionary & environmental sciences

For a reference copy of the document with all sections, see nature.com/documents/nr-reporting-summary-flat.pdf

Life sciences study design

All studies must disclose on these points even when the disclosure is negative.

Sample size	No sample size calculation was performed. For proteomics, phosphoproteomics and metabolomics, 4 different mouse embryonic stem cell lines were analysed, because the main purpose was to identify robust and consistent molecular changes across cell lines that possess different biological backgrounds. For the Rotenone sensitivity assay, two mouse embryonic stem cell lines were used, as the main purpose was to confirm the results derived from our proteomics data. The immunofluorescence of Erf was assessed in duplicates. For TEM, at least 10 cells were imaged per condition (two cell lines were analyzed). For Mito-tracker, two cell lines were used. Western blot for LC3-II was performed across 8 different days of 2i and Cdk8/19i treatment in one cell line.
Data exclusions	In the metabolomics study one of the samples was discarded in this step due to extremely low protein concentration and the high presence of missing values (cell line V6.4, treatment 2i, replicate 2).
Replication	Proteomics and phosphoproteomics analyses were performed using a label-based approach that minimizes experimental variation because samples are allocated in a multiplexed experiment and therefore adquired simultaneously in the LC-MS/MS system. Thus, for proteomics and phosphoproteomics, experiments were performed in four different mouse embryonic stem cell lines. On the other hand, metabolomics analyses were perfromed using a label-free approach. Thus, for metabolomics, four mouse embryonic stem cell lines were used including also four biological replicates from each cell line. Reproducibility of proteomics, phosphoproteomics and metabolomics was successful as determined by unsupervised clustering using Principal Component Analysis. For the Rotenone sensitivity assay, TEM of mitochondrial morphology, and mito-tracker, results were replicated in two independent mouse embryonic stem cells lines. The increased levels of LC3-II were confirmed in several days of 2i and Cdk8/19i treatments.
Randomization	For proteomics and phosphoproteomics, each cell line was analysed separately in a different isobaric labelling experiment. Batch-effects due to independent iTRAQ (proteomics) and TMT (phosphoproteomics) multiplexed-experiments were corrected using ComBat function in R. For metabolomics (performed by Metabolon, Inc), samples were randomized across the platform run with QC samples spaced evenly among the injection. For the Rotenone sensitivity assay, experimental groups (S/L, 2i and Cdk8/19i) and biological replicates (N=3) were analyzed in alternate order to minimize experimental biases.
Blinding	Researchers were not blinded to perform the experiments reported in this work as to keep a strict overview of sample labeling schemes, pre-fractionation and purification techniques as well as LC-MS/MS runs.

Reporting for specific materials, systems and methods

We require information from authors about some types of materials, experimental systems and methods used in many studies. Here, indicate whether each material, system or method listed is relevant to your study. If you are not sure if a list item applies to your research, read the appropriate section before selecting a response.

Materials & experimental systems

n/a	Involved in the study	
☐	☒	Antibodies
☐	☒	Eukaryotic cell lines
☒	☐	Palaeontology and archaeology
☒	☐	Animals and other organisms
☒	☐	Human research participants
☒	☐	Clinical data
☒	☐	Dual use research of concern

Methods

n/a	Involved in the study	
☒	☐	ChIP-seq
☐	☒	Flow cytometry
☒	☐	MRI-based neuroimaging

Antibodies

Antibodies used	Mu anti-ERF 1/250, Santa Cruz sc-398269<br>Goat anti-Mouse IgG (H+L) Highly Cross-Adsorbed Secondary Antibody, Alexa Fluor 488 from Invitrogen, Thermo Fisher Scientific, catalog # A-11029, RRID AB_2534088, Lot # 2179204. Antibody: Rabbit anti-LC3B, Cell Signaling #3868

	Goat anti-Rabbit IgG (H+L) Highly Cross-Adsorbed Secondary Antibody, Alexa Fluor 488 from Invitrogen, Thermo Fisher Scientific, catalog # A-11034, RRID AB_2576217, Lot #2256692.
Validation	The antibody has been previously employed for ERF detection in the following publication: "ERF deletion rescues RAS deficiency in mouse embryonic stem cells, Mayor-Ruiz, et al. 2018, Genes Dev. PMID: 29650524"

Eukaryotic cell lines

Policy information about cell lines

Cell line source(s)	Wild-type ES cells were derived at the Transgenic Mouse Unit of CNIO from E3.5 C57BL6 blastocysts (BL6), or mixed background C57BL6/129 (V6.4) blastocysts. Nanog-GFP knock-in mouse ES cells (TNGA, TON) were previously described by Chambers et al (Nature 2007) and were shared by the laboratory of Austin Smith. the ZS mouse ES line was a 2C-reporter shared by the laboratory of Minoru Ko. E14Tg2a.4 (wild-type parental, 129/Ola background) were from BayGenomics/ MMRRC resource, University of California
Authentication	The self-renewal properties and pluripotency markers of mouse ESCs were confirmed by RT-PCR, FACS, IF, RNASeq, proteomics and chimera developmental assays (see Lynch et al, PMID: 32989249).
Mycoplasma contamination	Cultures tested negative for mycoplasma.
Commonly misidentified lines (See ICLAC register)	No commonly misidentified cell lines were used in the current study.

Flow Cytometry

Plots

Confirm that:

- [x] The axis labels state the marker and fluorochrome used (e.g. CD4-FITC).
- [x] The axis scales are clearly visible. Include numbers along axes only for bottom left plot of group (a 'group' is an analysis of identical markers).
- [x] All plots are contour plots with outliers or pseudocolor plots.
- [x] A numerical value for number of cells or percentage (with statistics) is provided.

Methodology

Sample preparation	mouse Embryonic Stem Cells were treated with the indicated staining and then suspended in FACS Buffer before the analysis
Instrument	Samples were analyzed using a Gallios multi-color flow cytometrer instrument (Beckman Coulter, Inc, Fullerton, CA) set up with the 3-lasers 10 colors standard configuration
Software	Data was analyzed using FlowJo X
Cell population abundance	Cells used in this study were mouse embryonic stem cell lines kept in culture. No post-sort fractionation of any kind of subpopulation was required for this study
Gating strategy	Debris were excluded using SS-A vs FS-A, followed by gating on live cells using DAPI as a viability dye. Cells were then analyzed using the indicated fluorescent dyes. See gating strategy for live/dead selection in Suppl Fig 3C

- [x] Tick this box to confirm that a figure exemplifying the gating strategy is provided in the Supplementary Information.
